# Identifying the quality markers and optimizing the processing of Gastrodiae rhizoma to treat brain diseases

**DOI:** 10.3389/fphar.2024.1396825

**Published:** 2024-11-06

**Authors:** Yan Fu, Qingqing Xu, Jinqiang Zhang, Chuanzhi Kang, Changgui Yang, Lumei Guo, Chenggang Zhang, Tao Zhou, Chenghong Xiao

**Affiliations:** ^1^ Resource Institute for Chinese and Ethnic Materia Medica, Guizhou University of Traditional Chinese Medicine, Guiyang, China; ^2^ National Key Laboratory for Quality Insurance and Sustainable Use of Dao-di Herbs, National Resource Center for Chinese Materia Medica, Beijing, China; ^3^ Department of Chinese Medicine, Guizhou Institute of Food and Drug Control, Guiyang, China

**Keywords:** *Gastrodia elata* BL, fresh-cut processing, quality markers, neuroprotection, metabolomics, network pharmacology

## Abstract

**Background:**

Gastrodiae rhizoma (GR) refers to the dried tuber of *Gastrodia elata* Bl. and has been used for many centuries to treat brain diseases, such as Alzheimer’s disease, major depressive disorder, and cerebral ischemia. However, the processing of GR is complex and varied, resulting in unstable clinical treatment effects. The processing protocols significantly affect the active ingredients and curative effects of GR. We can optimize the processing of GR by identifying quality markers to treat brain diseases.

**Methods:**

Fresh tubers of *G. elata* Bl. were processed under eight different protocols, and their resulting contents of potentially bioactive compounds were compared using liquid chromatography mass spectrometry to screen the potential quality markers of GR through stoichiometric analysis. The potential quality markers of GR targeting Alzheimer’s disease, major depressive disorder, and cerebral ischemia were identified by network pharmacology, and the potentially neuroprotective effects of these components were validated through simulated docking to likely protein targets. Finally, a fit degree analysis was carried out using different composition ratios and proportions of the disease component degree value, and the therapeutic effects of different processing methods on Alzheimer’s disease, major depressive disorder, and cerebral ischemia were outlined clearly.

**Results:**

We identified 32 potential therapeutic components and screened 13 quality markers in GR, of which five quality markers (galactinol, glucosyringic acid, parishins C and E, and S-(4-hydroxybenzyl)-glutathione) showed efficacy against all three brain diseases. Furthermore, steaming and microwave-drying during processing can optimize the components of these quality markers for treating the three diseases.

**Conclusion:**

Processing protocols significantly affect the therapeutic components of GR and may also impact its effectiveness in treating brain diseases. Accordingly, optimizing the processing methods of GR to correspond to different therapeutic purposes may improve its efficacy against brain diseases.

## 1 Introduction

Diseases of the brain include acute brain injury with cerebral ischemia (CI) as well as chronic neurodegenerative conditions such as Alzheimer’s disease (AD) and major depressive disorder (MDD). These brain diseases are globally prevalent and can greatly reduce the quality of life. Most of the current drugs available against these conditions only relieve the symptoms and do not substantially slow the disease progression or recurrence. Hence, many people have sought traditional Chinese medicine (TCM) to treat these conditions with potentially fewer long-term side effects.

Gastrodiae rhizoma (GR) refers to the dried tubers of *Gastrodia elata* Bl. of the Orchidaceae family. It is a well-known component of TCM and a functional food used for many centuries to treat nervous-system-related diseases, including neurasthenia, migraine, and sciatica (Lu et al., 2023; Lu et al., 2022; Lin et al., 2021). The bioactive compounds found in GR are important in the management of brain diseases (El et al., 2024). Gastrodin has been reported to improve depression-like behavior and nerve cell damage in CUMS mice (Pei et al., 2024). GR has been shown to mitigate motor deficits and neuronal degeneration induced by G2019S mutations via glial Nrf2/Mad signaling, thereby averting the onset of Parkinson’s disease (Lin et al., 2021). Thus, GR can enhance recovery from CI by modulating the *in vivo* metabolic processes. It has been demonstrated that the therapeutic mechanisms of GR in the treatment of CI are associated with reduced inflammation, oxidative stress, neurotoxicity, and apoptosis (Wang et al., 2019).

The processing of GR is complex and varied, which has resulted in unstable clinical treatment effects (Kwon et al., 2014). Most of the GR are steamed during processing, except for a few. At the same time, the drying methods of GR include drying under the sun, with hot air, by freezing, or with microwaves (Wu et al., 2022). In fact, several studies have revealed that these differences in the drying methods can affect the content and proportion of the medicinal ingredients in Chinese medicinal materials, thereby affecting their therapeutic efficacies (Li et al., 2019; Xie et al., 2023; Wu et al., 2022; Su et al., 2024). The differences in the chemical compositions of GR are mainly with respect to the phenolic and parishin components. Fresh GR (FGR) extract has the highest content of p-hydroxybenzyl alcohol. The parishin contents of dried GR processed products were higher than those in FGR. Recently, there is growing popularity of GR processing in Asian countries with the implementation of policies allowing its fresh processing; this not only reduces time and costs but also holds potential curative value. The extensive use of GR in the treatment of brain diseases as well as the complexity and diversity of the regulations over GR processing protocols may be the main factors for the unstable clinical therapeutic effects of GR. Nevertheless, there is a paucity of research on whether variations in the treatment outcomes for brain diseases can be attributed to the different processing methods of GR. We hypothesized that the quality markers of GR used in the treatment of different brain diseases could be identified such that the processing methods of GR could be optimized according to the therapeutic purpose and components of the corresponding quality markers to improve the therapeutic efficacy and clinical stability of GR.

Herein, we compared the contents of potentially therapeutic compounds in GR after the application of various processing protocols. Using network pharmacology and metabolomics, we predicted several compounds that were most likely to be bioactive against three disorders, namely, AD, MDD, and CI, and used these “quality markers” to identify the processing protocols that are more appropriate for preparations targeting each of the disorders.

## 2 Materials and methods

### 2.1 Processing protocols

FGR was collected from Bijie in Dafang county of Guizhou Province, China. The fresh tubers were sliced and then dried under the sun (SD), in hot air at 60°C (HD), by freezing (FD), or in a microwave (MD). Alternatively, the fresh tubers were sliced, steamed for 20 min, and then dried under the sun (Steam-SD), in hot air at 60°C (Steam-HD), by freezing (Steam-FD), or in a microwave (Steam-MD). Three batches of tubers were processed each according to these eight protocols.

### 2.2 Analysis of potentially therapeutic compounds in the preparations

Herbal preparations formulated as described in Section 2.1 were analyzed by liquid chromatography mass spectrometry. The test solution was prepared by weighing 0.25 g of GR powder accurately, followed by addition of 10 mL of 50% ethanol, ultrasonic extraction for 40 min, and transfer to a centrifuge tube; the sample was then centrifuged at 10,000 r/min for 10 min, and repeated extraction was performed once, followed by combining the filtrate and passing through a 0.22-µm filter.

The samples were individually injected onto the HSS T3 column (2.1 mm × 100 mm, 1.8 μm) on an Acquity ultrahigh-performance liquid chromatography system (Waters, Manchester, United Kingdom). Separation was then performed using a gradient of (A) 0.1% formic acid in water and (B) 0.1% formic acid in acetonitrile as follows: linear gradient of 0%–0.5% of B from 0 to 4 min, 0.5%–2% of B from 4 to 6 min, 2%–8% of B from 6 to 7 min, 8%–12% of B from 7 to 12 min, 12%–20% of B from 12 to 18 min, 20%–40% of B from 18 to 24 min, 40%–45% of B from 24 to 25 min, 45%–70% of B from 25 to 31 min, 70%–98% of B from 31 to 33 min, and 98% of B from 33 to 35 min. Throughout the procedure, the column temperature was maintained at 40°C and flow rate was maintained at 0.3 mL/min.

The eluent from the column was placed in a Xevo G2-XS high-definition quadrupole time-of-flight mass spectrometer (Q-TOF MS; Waters) equipped with an electrospray ionization source. Mass spectrometry was then performed in the negative ion mode with the following parameters: capillary voltage of 3.0–2.5 kV; sample cone voltage of 3 V; source temperature of 120°C; desolvation temperature of 500°C; nitrogen flow rate of 900 L/h; cone gas flow rate of 10 L/h; rate of TOF acquisition of 0.3 s/scan. Data were also collected in the centroid mode over the mass range of *m/z* of 50–1,500 (Lai et al., 2017).

### 2.3 *In silico* prediction of the potential therapeutic components in GR and their disease targets

The potential therapeutic components in GR and their disease targets were predicted using PharmMapper (www.lilab-ecust.cn/pharmmapper), SEA (https://sea.bkslab.org), and the Swiss Target Prediction and Analysis Platform (http://swisstargetprediction.ch) after removing the duplicate genes. The potential disease targets were mapped to gene names using the UniProt database (www.uniprot.org) and checked for overlap against the disease targets in AD, MDD, and CI in the GeneCards (www.genecards.org) and OMIM (Shi et al., 2023) databases. Only the targets in the species *Homo sapiens* were analyzed, and only those targets with values of “betweenness centrality,” “closeness centrality,” and “degree centrality” exceeding the software-specified reference values based on the network of protein–protein interactions generated in String (https://string-db.org) and visualized using Cytoscape 3.6.1 (Zhou et al., 2023) were retained in the final analysis. Enrichment of the genes encoding potential disease targets in the gene ontology and Kyoto encyclopedia of genes and genomes (KEGG) pathways was analyzed using the Metascape database (https://metascape.org) (Zhou et al., 2019). Twenty KEGG pathways for which enrichments were associated with *p* < 0.05 were selected and sorted in ascending order of *p* value. The networks of potential disease targets and pathways were then constructed using Cytoscape.

### 2.4 Molecular docking of the potential therapeutic components in GR with their predicted disease targets

The potential therapeutic components in GR were docked to potential disease targets with degrees ≥20 in the networks derived as above. The three-dimensional chemical structures of the core active ingredients in GR were obtained from the PubChem database (NCBI, United States), and minimized for energy using molecular mechanics-2 (MM2) force fields in Chem 3D Ultra 14.0 (Cambridge Soft Corporation, Boston, MA, United States). The RCSB Protein Data Bank (www.rcsb.org) was used to retrieve the crystal structures of the target proteins, and AutoDock Tools 1.5.7 (Center for Computational Structure Biology, La Jolla, CA, United States) was used to add the hydrogen atoms and remove the water and heterogeneous molecules; AutoDock vina was then employed to predict potential molecular binding patterns between the components and candidate targets. The docked structures were analyzed via PyMol 2.3.0 (Schrodinger, New York, NY, United States) (Zhong et al., 2023).

### 2.5 *In silico* optimization of processing of GR to treat specific diseases

The term “degree” in this study refers to the number of node connections in the entire network that reflect the interactions between the nodes, and it is proportional to the importance of the core goal (Liu et al., 2021). The potential therapeutic components in GR with degrees ≥10 in the networks were defined as “quality markers,” whose levels in the herbal preparations could be used to predict the therapeutic efficacies against certain diseases (Gonzalez-Juarez et al., 2020). We focused on three disorders, namely, AD, MDD, and CI. For each condition, components with degrees ≥10 were considered as the quality markers, and the total degree from combining all quality marker values was normalized to yield a proportion for that marker. The proportions of each of the quality markers for the eight processing protocols for each of the three diseases were analyzed using principal component analysis in Simca-p 14.1 (Umetrics, Umea, Sweden) to reveal the clustering of processing methods and diseases. The clustering was further refined using orthogonal partial least-squares discriminant analysis (Zhou et al., 2023). The fitting degrees were calculated as described to compare the predicted therapeutic efficacies of each of the processing protocols against each of the diseases.

### 2.6 Other statistical methods

The data distribution was evaluated using the Kolmogorov–Smirnov test in GraphPad Prism 9.5. For data with normal distributions, one-way ANOVA was used to analyze the differences between groups; the Kruskal–Wallis test was used for data with non-normal distributions. The significance level used was *p* < 0.05 (*), and the data were represented as means ± standard errors of the means (SEMs).

## 3 Results

### 3.1 Various processing methods resulted in different levels of 46 potential therapeutic components in GR

**FIGURE 1:**
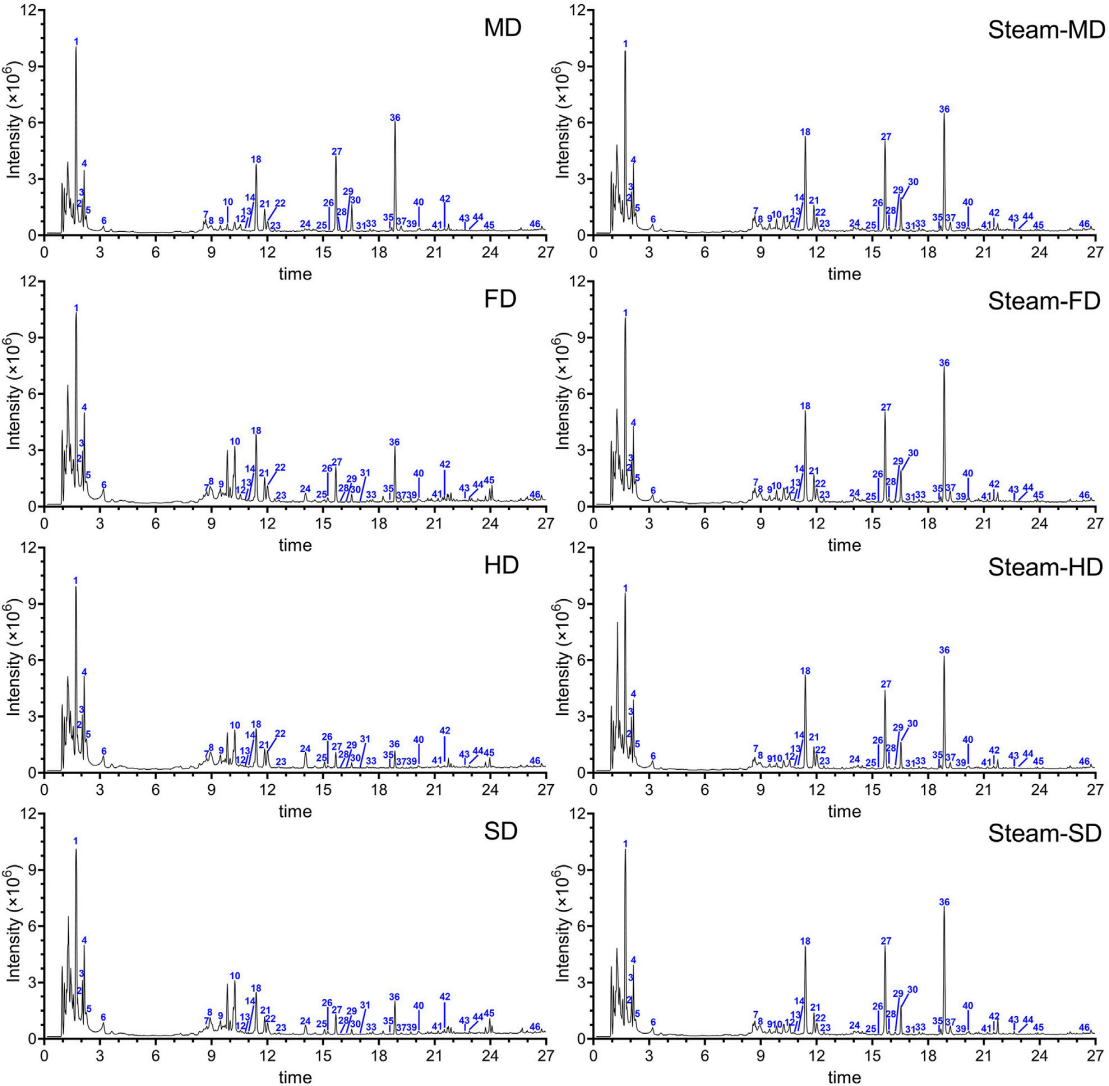
Representative total ion chromatograms of GR after applying the eight processing protocols. SD: Fresh tubers were sliced and dried under the sun. HD: Fresh tubers were sliced and dried with hot air at 60°C. FD: Fresh tubers were sliced and dried by freezing. MD: Fresh tubers were sliced and dried in a microwave. Steam-SD: Fresh tubers were sliced, steamed, and dried under the sun. Steam-HD: Fresh tubers were steamed and dried with hot air at 60°C. Steam-FD: Fresh tubers were steamed and dried by freezing. Steam-MD: Fresh tubers were steamed and dried in a microwave.

A total of 46 signal peaks were detected, for which 32 compounds were characterized in GR; these included 21 aromatic hydrocarbons, five organic acids, and six heteroatomic compounds (comprising three nitrogen and three sulfur compounds) (Figure 1; Supplementary Table S1). Aromatics, phenolics, and polysaccharides have long been considered the most likely bioactive components (Shan et al., 2021). We identified 4,4′-dihydroxydibenzyl ether, which is one of the polybenzyl compounds previously identified in the tuber (Li et al., 2019). We also detected nearly all parishins that were previously found in the tuber; these compounds show several biological and pharmacological effects (Zeng et al., 2023). We identified several nitrogen- and sulfur-containing compounds that were previously reported in GR (Yu et al., 2022), including nitrogen-containing grossamide that is known to act against neuroinflammation (Luo et al., 2017) and the S-containing compounds 4,4′-dihydroxybenzyl sulfoxide, S-(4-hydroxybenzyl)-glutathione, and S-(gastrodin)-glutathione. We found that different processing methods resulted in different levels of 46 potential therapeutic components in GR. For example, S-(4-hydroxybenzyl)-glutathione levels were substantially higher in MD preparations with or without steaming or in preparations that had been steamed and freeze-dried. Among the organic acids detected were the derivatives of glucosyringic and citric acids. The levels of citric acid were higher in HD preparations. These results suggest that the processing method can significantly affect the level of active ingredients in GR.

### 3.2 Identification of the quality markers in GR after processing

**FIGURE 2:**
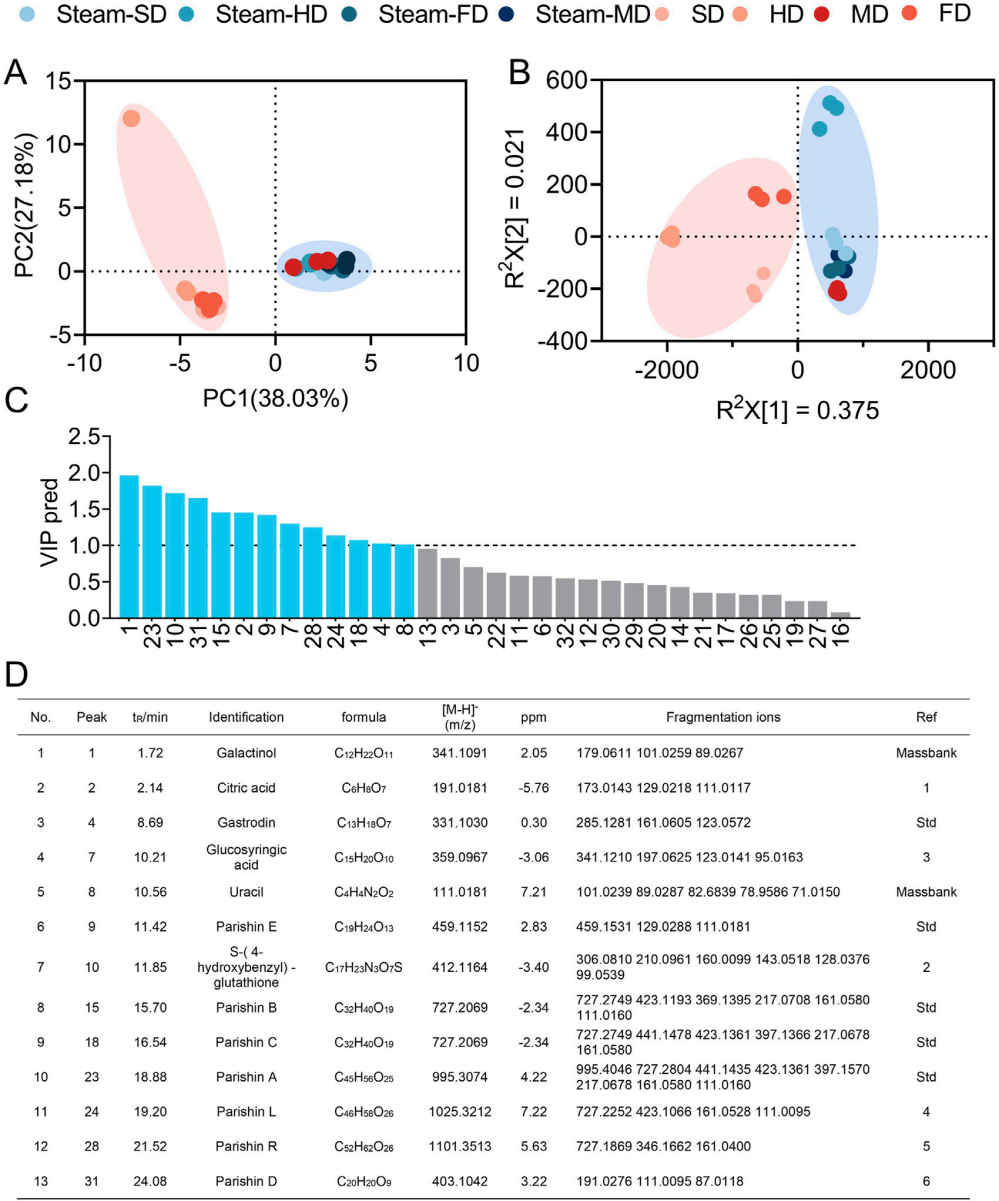
Clustering of GR preparations according to processing protocols based on the quality markers. Clustering based on **(A)** principal component analysis and **(B)** orthogonal partial least-squares discriminant analysis. **(C, D)** Identification of the quality markers. PC, principal component; VIP pred, predicted value of the variable importance in projection. **(D)** References are shown in Supplementary Table S1.

Using a combination of principal component and partial least-squares discriminant analyses, we found that the preparations of GR were clustered according to the processing protocols (Figures 2A, B), indicating that the complement of potentially bioactive components in the herbal preparations differed substantially based on steaming and the drying methods used. We identified 13 potential quality markers based on variable importance among the projected values, retention times, and molecular masses (Figures 2C, D), namely, galactinol, citric acid, gastrodin, glucosyringic acid, S-(4-hydroxybenzyl)-glutathione, uracil, parishins A–E, parishin L, and parishin R.

**FIGURE 3:**
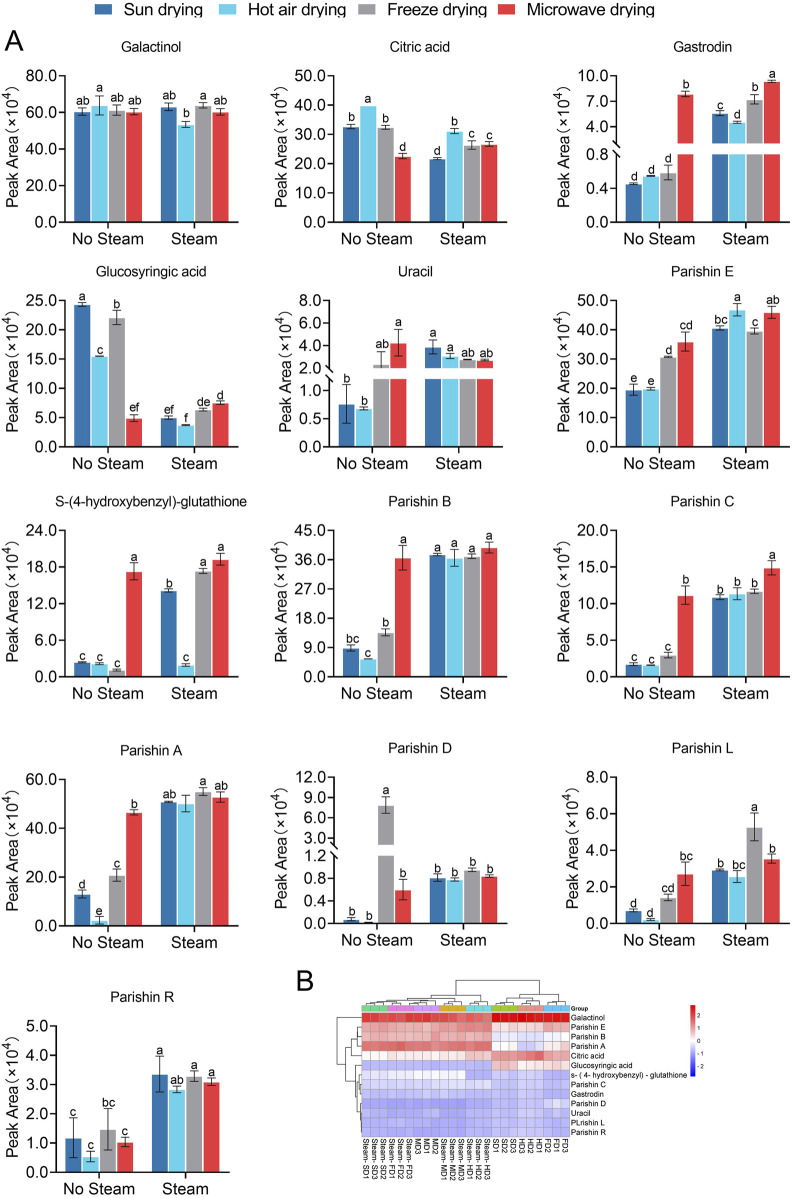
Comparison of the levels of 13 quality markers in GR after applying the eight processing protocols. **(A)** Bar plots where bars with different letters differ significantly from each other (*p* < 0.05). **(B)** Heatmap showing the clustering of quality markers (rightmost column) and processing protocols (along the bottom of each column). Results are shown for three batches subjected to each protocol (marked as “1”, “2”, and “3”). Different superscript letters indicate significant differences (*p* < 0.05). The statistical analysis depicted in **(A)** entails one-way analysis of variance (Supplementary Table S3), and the normal distribution test is shown in Supplementary Table S2.

Taking the ion peak areas as representatives of the levels of the parental components in the herbal preparations (Tong et al., 2019), we compared the 13 quality markers for the various processing methods. Regardless of the drying method used, steaming resulted in higher levels of gastrodin as well as parishins A–C and E but lower levels of glucosyringic acid (Figure 3A). Among the samples that were not steamed, microwave drying resulted in higher levels of gastrodin, S-(4-hydroxybenzyl)-glutathione, uracil, as well as parishins A–C and E but lower levels of glucosyringic acid compared to the other drying methods. Among the samples that were steamed, the quality marker levels generally did not differ between freeze-drying and microwave drying but S-(4-hydroxybenzyl)-glutathione levels were significantly lower after hot-air drying (Figure 3A). The 13 quality markers showed two distinct behavioral profiles across the eight processing protocols: glucosyringic acid, S-(4-hydroxybenzyl)-glutathione, parishin C, gastrodin, parishin D, uracil, parishin L, and parishin R behaved similar to each other, whereas the remaining five components showed unique behaviors (Figure 3B). Accordingly, the marker profiles were similar for the three protocols that involved drying under the sun, with hot air, or by freezing without prior steaming, whereas the marker profiles for the other five protocols were similar to each other.

### 3.3 Identification of the quality markers in GR to target different brain diseases

**FIGURE 4:**
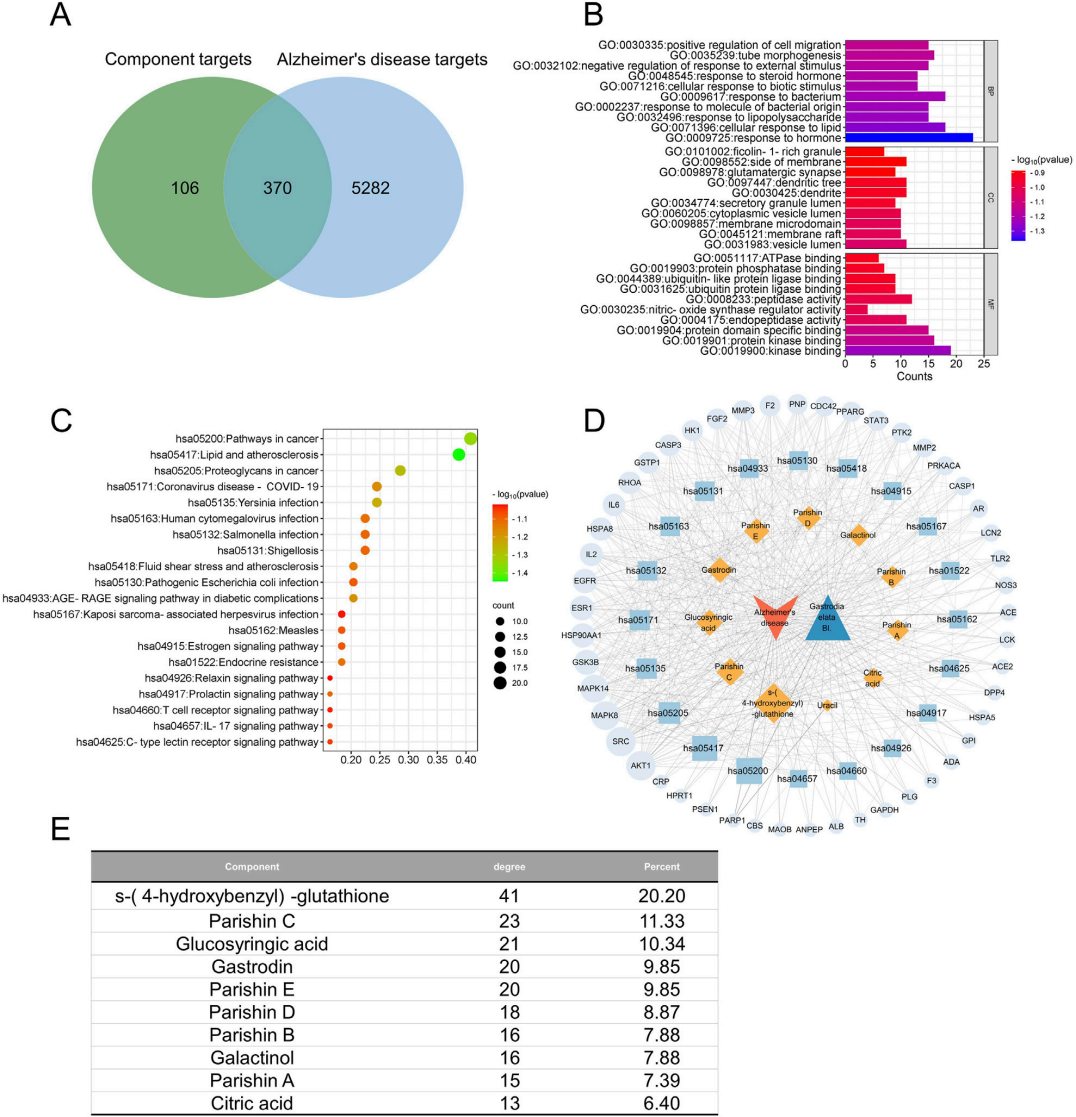
Network pharmacology of the quality markers in GR after processing and their potential protein targets in Alzheimer’s disease. **(A)** Overlap between proteins potentially targeted by 10 quality markers and those potentially associated with the disease. **(B)** Enrichment of the potential targets in the gene ontology biological processes (BPs, top), cellular compartments (CCs, middle), and molecular functions (MFs, bottom). **(C)** Enrichment of the potential targets in the Kyoto Encyclopedia Of Genes And Genomes pathways. **(D)** Network of interactions among the quality markers and potential protein targets. **(E)** Quality markers showing the highest degrees of connectedness in the network in **(D)**.

**FIGURE 5:**
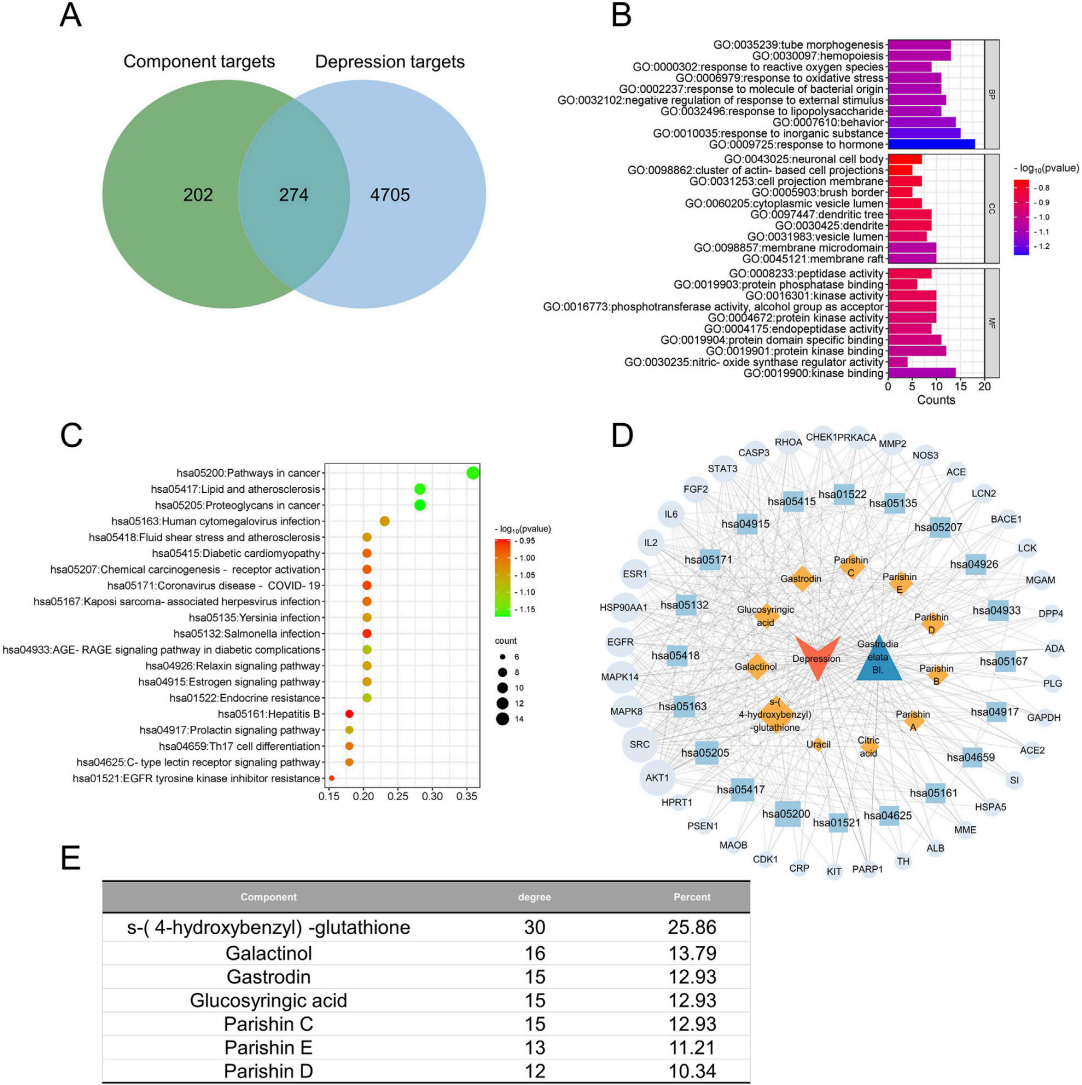
Network pharmacology of the quality markers in GR after processing and their potential protein targets in major depressive disorder. **(A)** Overlap between proteins potentially targeted by 7 quality markers and those potentially associated with the disease. **(B)** Enrichment of the potential targets in the gene ontology biological processes (BPs, top), cellular compartments (CCs, middle), and molecular functions (MFs, bottom). **(C)** Enrichment of potential targets in the Kyoto encyclopedia of genes and genomes pathways. **(D)** Network of interactions among the quality markers and potential protein targets. **(E)** Quality markers showing the highest degrees of connectedness in the network in **(D)**.

**FIGURE 6:**
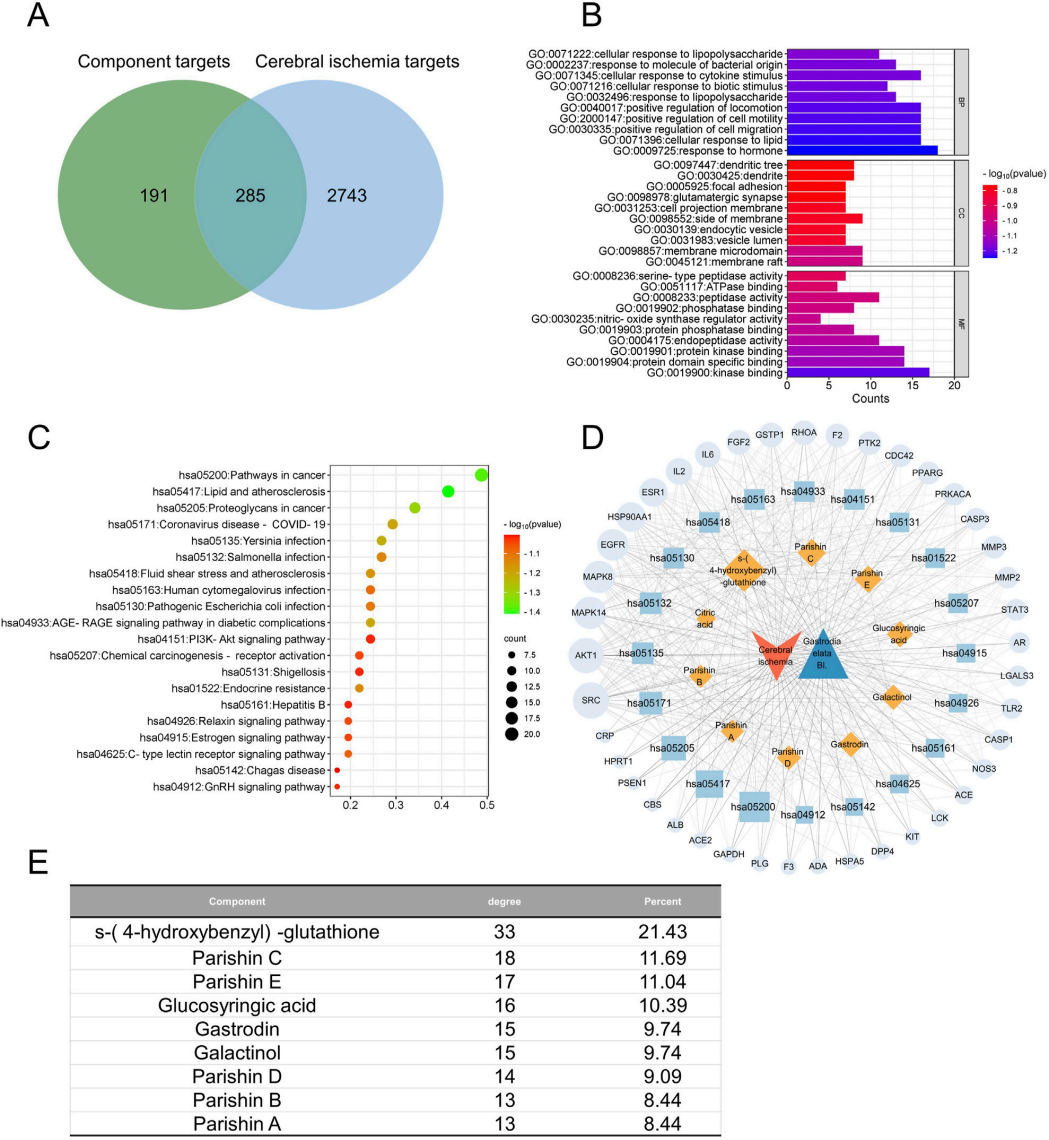
Network pharmacology of the quality markers in GR after fresh-cut processing and their potential protein targets in cerebral ischemia. **(A)** Overlap between proteins potentially targeted by 9 quality markers and those potentially associated with the disease. **(B)** Enrichment of the potential targets in the gene ontology biological processes (BPs, top), cellular compartments (CCs, middle), and molecular functions (MFs, bottom). **(C)** Enrichment of potential targets in the Kyoto Encyclopedia Of Genes And Genomes pathways. **(D)** Network of interactions among the quality markers and potential protein targets. **(E)** Quality markers showing the highest degrees of connectedness in the network in **(D)**.

The PubChem database revealed a total of 476 proteins that could potentially be targeted by 11 of the quality markers; we excluded parishins R and L from this analysis as we were unable to identify any potential targets for their binding. In the GeneCards and OMIM databases, we identified 5,652 or 4,979 or 3,028 proteins that were potentially targeted by one of the quality markers related to AD or MDD or CI, respectively; accordingly, we screened 370, 274, and 285 proteins for AD, MDD, and CI that may be targeted by the 11 quality markers in GR (Figures 4A, 5A, 6A).

In AD, the 370 potential targets were enriched in biological processes related to upregulation of cell migration, tubular morphogenesis, downregulation of responses to external stimuli, and responses to steroid hormones (Figure 4B). These targets were enriched in the following cellular compartments: granules rich in ficolin-1, one side of the membrane, glutamatergic synapses, and dendritic trees. These were enriched in the molecular functions of binding to ATPases, protein phosphatases, ubiquitin protein ligases, and similar ligases. The targets were enriched in the KEGG signaling pathways related to cancer, proteoglycans in cancer, lipids and atherosclerosis, coronavirus disease, and *Yersinia* infections (Figure 4C). Integration of all these results allowed the identification of the 10 most relevant therapeutic components of GR in the treatment of AD, namely, S-(4-hydroxybenzyl)-glutathione, parishins A–E, glucosyringic acid, galactinol, gastrodin, and citric acid. Accordingly, the most relevant disease targets were the proteins encoded by the genes *AKT1*, *SRC*, *MAPK8*, *MAPK14*, and *GSK3B* (Figures 4D, E).

In MDD, the 274 potential targets were enriched in biological processes related to tubular morphogenesis, hematopoiesis, responses to active oxygen species, and responses to oxidative stress (Figure 5B). The targets were enriched in the following cellular compartments: neuronal cell bodies, clusters of actin-based cell projections, cell projection membranes, and brush borders. These were enriched in the molecular functions of peptidase activity, protein phosphatase binding, kinase activity, phosphotransferase activity, and alcohol group as the acceptor. The targets were enriched in the KEGG signaling pathways related to cancer, lipids and atherosclerosis, proteoglycans in cancer, and human cytomegalovirus infection (Figure 5C). Integration of all these results allowed us to identify the seven most relevant therapeutic components of GR in the treatment of MDD, namely, S-(4-hydroxybenzyl)-glutathione, galactinol, gastrodin, glucosyringic acid, and parishins C–E. Accordingly, the most relevant disease targets were the proteins encoded by the genes *AKT1*, *SRC*, *MAPK8*, *MAPK14*, *HSP90AA1*, *EGFR*, and *ESR1* (Figures 5D, E).

In CI, the 285 potential targets were enriched in biological processes related to lipopolysaccharides, responses to molecules of bacterial origin, cellular responses to cytokine stimuli, and cellular responses to biotic stimuli (Figure 6B). The targets were enriched in the following cellular compartments: dendritic tree, dendrites, focal adhesion, and glutamatergic synapses. These were enriched in the molecular functions of serine-type peptidase activity, ATPase binding, peptidase activity, and phosphatase binding. The targets were enriched in the KEGG signaling pathways related to cancer, lipids and atherosclerosis, and proteoglycans in cancer (Figure 6C). Integration of all these results allowed us to identify the nine most relevant therapeutic components of GR in the treatment of CI, namely, S-(4-hydroxybenzyl)-glutathione, parishins A–E, glucosyringic acid, galactinol, and gastrodin. Accordingly, the most relevant disease targets were the proteins encoded by the genes *SRC*, *AKT1*, *MAPK8*, *MAPK14*, and *EGFR* (Figures 6D, E).

**FIGURE 7:**
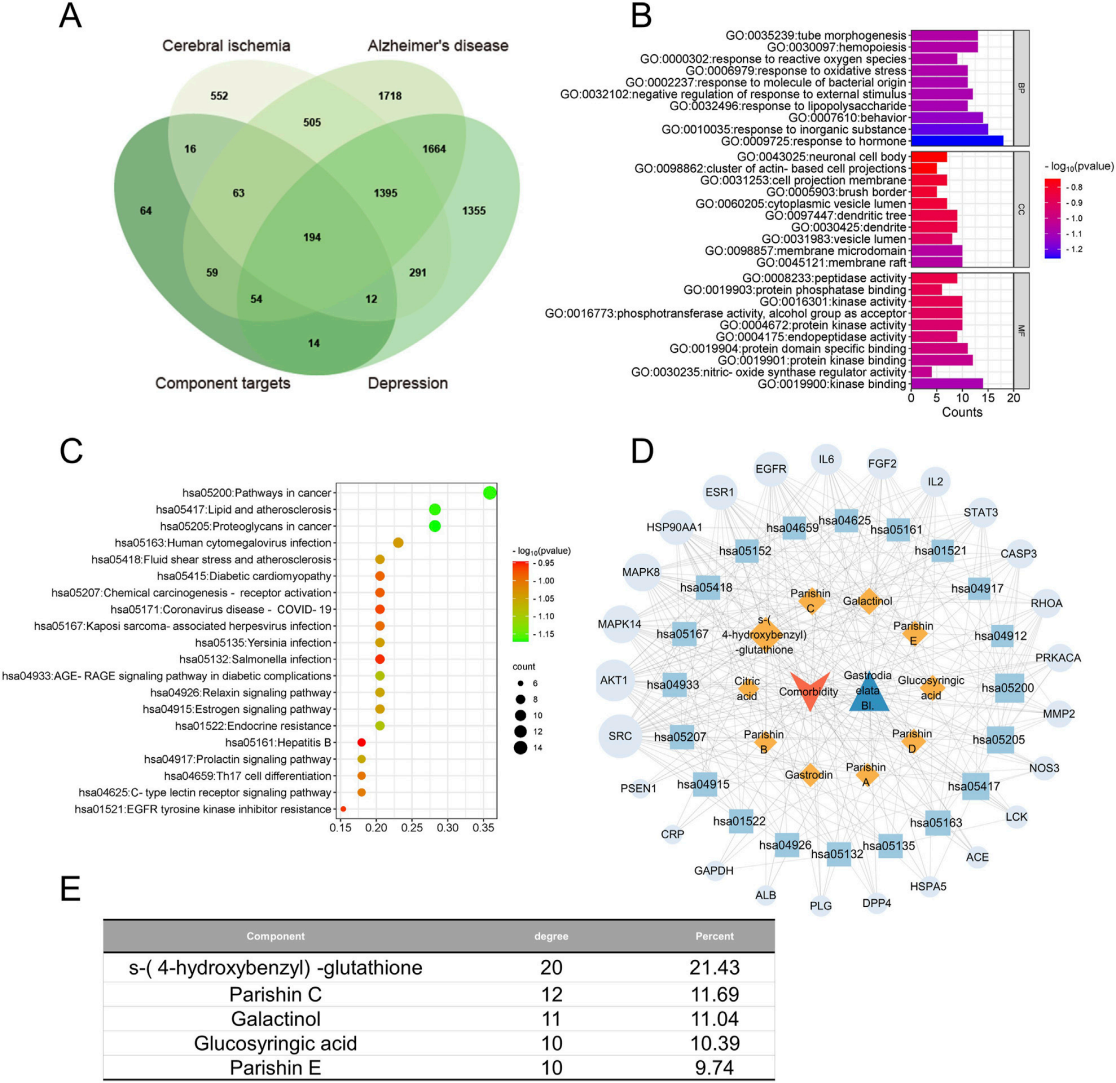
Network pharmacology of the quality markers in GR after fresh-cut processing and the potential protein targets simultaneously related to Alzheimer’s disease, major depressive disorder, and cerebral ischemia. **(A)** Overlap between the component targets and potential protein targets related to each of the diseases. **(B)** Enrichment of the potential targets in the gene ontology biological processes (BPs, top), cellular compartments (CCs, middle), and molecular functions (MFs, bottom). **(C)** Enrichment of potential targets in the Kyoto Encyclopedia Of Genes And Genomes pathways. **(D)** Network of interactions among the quality markers and potential protein targets. **(E)** Quality markers showing the highest degrees of connectedness in the network in **(D)**.

Among all the predicted protein targets across the three disorders, we identified 194 that were common to all of them (Figure 7A). These 194 targets were enriched in the KEGG pathways related to cancer, lipids and atherosclerosis, proteoglycans in cancer, and human cytomegalovirus infection (Figures 7B, C). Integration of these results allowed us to identify five components of GR that were most relevant to treating cooccurrences of the three diseases, namely, S-(4-hydroxybenzyl)-glutathione, parishins C and E, glucosyringic acid, and galactinol; accordingly, the most relevant disease targets were the proteins encoded by the genes *AKT1*, *SRC*, *MAPK8*, *MAPK14*, *HSP90AA1*, and *EGFR* (Figures 7D, E).

### 3.4 *In silico* validation that the quality markers in GR can bind to the targets of neuropsychiatric diseases

**FIGURE 8:**
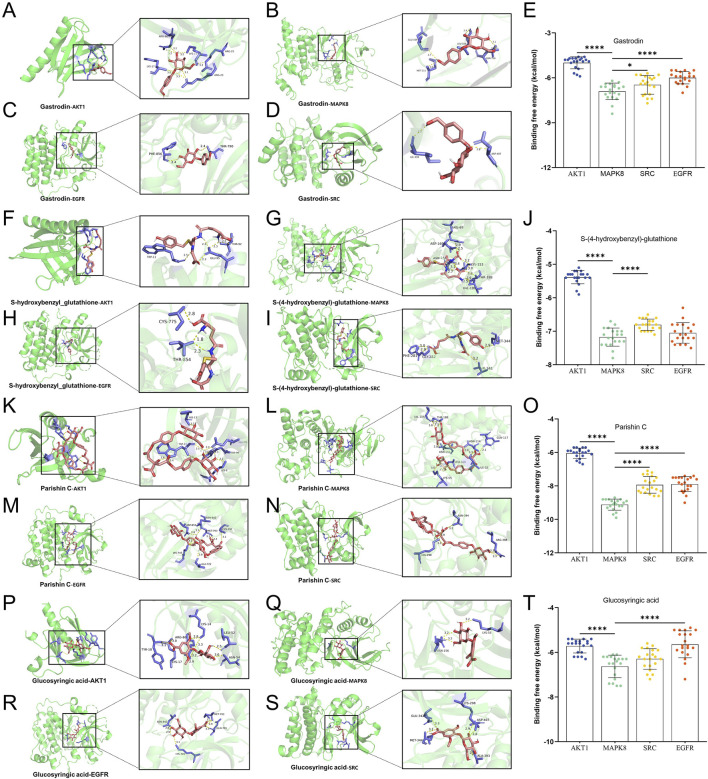
Molecular docking simulations of the quality markers in GR after processing and the potential protein targets simultaneously related to Alzheimer’s disease, major depressive disorder, and cerebral ischemia. Molecular dockings of **(A–D)** gastrodin with *AKT1*, *MAPK8*, *SRC*, and *EGFR*; **(F–I)** S-(4-hydroxybenzyl)-glutathione with *AKT1*, *MAPK8*, *SRC*, and *EGFR*; **(K–N)** glucosyringic acid with *AKT1*, *MAPK8*, *SRC*, and *EGFR*; **(P–S)** parishin C with *AKT1*, *MAPK8*, *SRC*, and *EGFR.* **(E, J, O, T)** Binding energy table of the key components and key targets. The simulated complexes are shown as ribbon diagrams, while the herbal components and side chains in the target proteins predicted to interact with them are shown as ball-and-stick models. Amagnified view of the binding site is shown to the right of each complex. The bar plots compare binding energies for the complex of each herbal component with each protein target. *p < 0.05, ****p < 0.001. The statistical analysis whose results are depicted in **(E, J, O, T)** entails a one-way analysis of variance (Supplementary Table S5), and the normal distribution test is shown in Supplementary Table S4.

We assessed the potential for the components of GR to interact with their predicted protein targets through molecular docking simulations. Thus, we focused on the components and targets common to the three brain diseases. These simulations predicted that the four components gastrodin, S-(4-hydroxybenzyl)-glutathione, parishin C, and glucosyringic acid could form complexes with binding energies below −5 kcal/mol with the proteins encoded by the following genes: *SRC*, *AKT1*, *MAPK14*, *EGFR*, *HSP90AA1*, and *ESR1* (Figure 8). The simulations predicted the lowest binding energies and hence most stable bindings for the following complexes: gastrodin with *MAPK8*, S-(4-hydroxybenzyl)-glutathione with *MAPK8*, parishin C with *MAPK8*, and glucosyringic acid with *MAPK8*.

### 3.5 Screening the optimal processing protocol for different brain diseases

We estimated the extent to which each of the quality markers in GR contributed to the total degree of predicted therapeutic efficacy against each of the three brain diseases, and we then compared these contributions across the eight processing protocols. Our goal was to determine whether certain protocols may be better in terms of the fitting degree for preparing the tubers as treatments against the given diseases.

**FIGURE 9:**
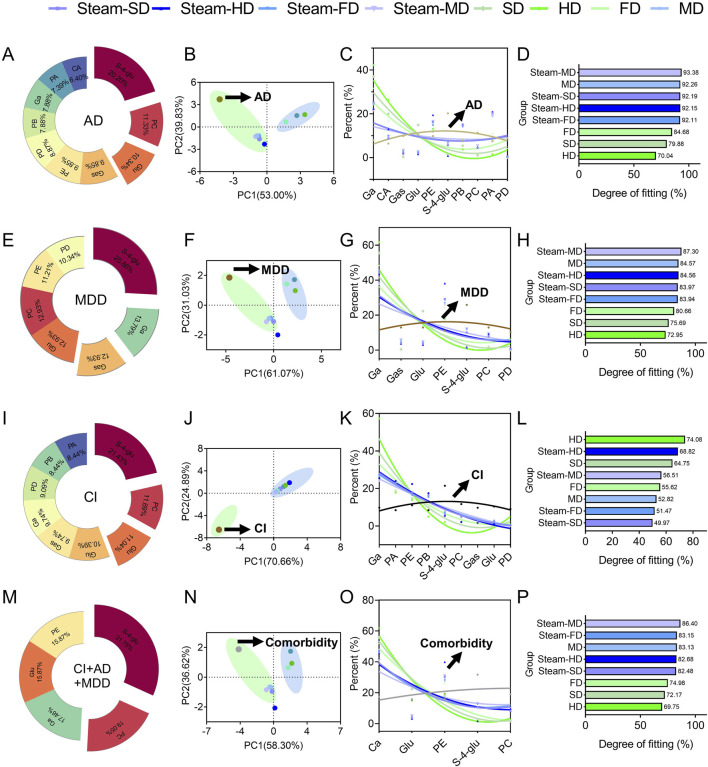
Analysis of fit degree. *In silico* predictions of the optimal processing protocols to prepare GR against **(A–D)** Alzheimer’s disease, **(E–H)** major depressive disorder, **(I–L)** cerebral ischemia, or **(M–P)** all three conditions simultaneously. In each row, the first panel shows the contributions of the individual quality markers to the total degree of the network of interactions among the quality markers and potential protein targets; the second panel shows the results of principal component analysis to detect the clusters of processing protocols; the third panel shows the fitting curve; the fourth panel shows the fitting degree assigned to each protocol.

Ten of the quality markers, namely, galactinol, citric acid, gastrodin, glucosyringic acid, parishins A–E, and S-(4-hydroxybenzyl)-glutathione), contributed substantially to the predicted therapeutic efficacy against AD (Figure 9A); principal component analysis of the variations in the contributions across the processing protocols showed several clusters (Figure 9B), among which the following four showed the highest fitting degrees: steaming followed by any type of drying except freeze-drying, and microwave drying without prior steaming (Figures 9C, D). These results suggest that exposure to high temperatures during processing could improve the efficacy against AD.

Seven of the quality markers, namely, galactinol, gastrodin, glucosyringic acid, parishin E, S-(4-hydroxybenzyl)-glutathione, parishin C, and parishin D, contributed substantially to the predicted therapeutic efficacy against MDD (Figure 9E). Based on the results of principal component analysis of the contributions of the different processing methods, four treatments were associated with MDD: steam-SD, steam-FD, steam-MD, and MD (Figure 9F); among these treatments, Steam-HD showed the best fit (Figures 9G, H). These results indicate that the Steam-MD and MD methods were most associated with the symptoms of MDD, suggesting that microwave drying in the preparation of the GR decoction pieces could potentially offer better treatment outcomes (Figure 9H).

Nine of the quality markers, namely, galactinol, parishin A, parishin E, parishin B, S-(4-hydroxybenzyl)-glutathione, parishin C, gastrodin, glucosyringic acid, and parishin D, contributed substantially to the predicted therapeutic efficacy against CI (Figure 9I); through principal component analysis of the contributions of the different processing methods, it was found that CI was not clustered with any of the processing methods (Figure 9J). However, drying under the sun without steaming showed the best fit for CI (Figures 9K, L). The fitting analysis results showed that hot-air drying of GR had the highest similarity to CI among all tested processing methods; this implies that hot-air drying as a processing method of GR may offer better treatment potential for patients with CI (Figure 9L).

Finally, we focused on the quality markers that were likely to show efficacy against all three brain diseases, namely, galactinol, glucosyringic acid, parishins C and E, and S-(4-hydroxybenzyl)-glutathione (Figures 9M–P). We identified the best processing protocols as steaming followed by drying with hot air or in a microwave as well as microwave drying without prior steaming.

## 4 Discussion

In this study, we compared the profiles of potentially bioactive compounds in GR after applying various processing protocols. We used *in silico* methods to identify certain herbal components that can serve as quality markers in the preparations designed to treat three brain diseases, namely AD, MDD, and CI. Based on the relative levels of these markers, we show that certain processing protocols may be better than others for producing GR against a given disease. The profiles of the chemicals that we detected in the herbal preparations are consistent with those reported previously for *G. elata* (Su et al., 2023). We identified several components after fresh-cut processing of GR beyond those reported by other investigators after processing the tubers using the “Jianchang Bang” method, which involves steaming and drying in the presence of ginger juice (Ye et al., 2019). In particular, we detected more organic acids as well as gastrodin, *p*-hydroxybenzyl alcohol, and other phenolics that were not reported previously. Nevertheless, we detected fewer varieties of organic acids than the extant reports after processing fresh and dried immature/mature stem tubers (Lai et al., 2017) along with more phenolics and parishins.

We identified several quality markers of GR through *in silico* analysis that predicted the potential effects against AD, MDD, and/or CI. Some of these markers have already been linked to neuroprotective activities, implying the reliability of our *in silico* findings. parishins show antiepileptic, anticonvulsive, sedative, and neuroprotective effects (Zeng et al., 2023), and parishin C has been shown to inhibit the release of oxidative stress and pro-inflammatory factors in a rat model of cerebral ischemia, mitigating tissue injuries and protecting neural functions. S-(4-hydroxyphenyl)-glutathione and potentially the other components of GR may protect the neurons against excitotoxicity involving glutamate receptors (Andersson et al., 1995) as well as hyperactivation and apoptosis of the microglia (Hsieh et al., 2005). Our docking studies predict that gastrodin, parishin C, S-(4-hydroxyphenyl)-glutathione, and glucosyringic acid can bind to MAP kinase 14 and Akt kinases; activation of these enzymes and their signaling pathways exert neuroprotective effects in different contexts, including neurodegenerative diseases (Li et al., 2022; Song et al., 2023). These simulations are consistent with experimental findings that gastrodin inhibits MAP kinase signaling and hence hyperactivation of the microglia that is associated inflammatory responses and seizures in an animal model of epilepsy (Tao et al., 2023). The docking studies further predicted that four of the tuber components could bind to ESR1 and HSP90, both of which serve as downstream effectors of the signaling pathways involving MAP and Akt kinases (Stirone et al., 2005); they also bind to Src kinase that activates signaling involving MAP kinases and whose inhibition can exert neuroprotective effects (Hiscox et al., 2006). Our *in silico* analyses identified several proteins and signaling pathways that can be investigated in future research to clarify and optimize the therapeutic efficacy of GR against major brain diseases.

We found that the quality marker levels differed depending on the processing protocol used. This was particularly noticeable for gastrodin, S-(4-hydroxybenzyl)-glutathione, and the parishins B, C, and E; the levels of these components were generally higher when the tubers were steamed before drying. Previous studies have shown that steaming the tubers can increase the levels of gastrodin, *p*-hydroxybenzyl alcohol, *p*-hydroxybenzaldehyde, and parishins A, B, and E (Wu et al., 2022). These findings reflect that steaming inhibits hydrolases (Li et al., 2019). Indeed, steaming increases the content of gastrodin and prevents the browning of *G. elata* during drying by inactivating the polyphenol oxidase enzymes that often trigger enzymatic browning reactions. Steaming is an essential operation during processing and is vital for quality formulation (Xie et al., 2023). Of the eight processing protocols assessed herein, our chemical and *in silico* analyses suggest that the following five are better suited to preparing GR for individuals with brain diseases: steaming followed by any of the four drying methods or microwave drying without prior steaming. AD and MDD showed the highest correlations with Steam-MD, while CI exhibited the strongest association with HD. Microwave drying was associated with a higher fitting degree than the other drying methods; this drying method also has the added advantage of sterilizing the herbal material and has shown benefits in the preparation of other TCMs rich in volatile oils, polysaccharides, or saponins (Qin et al., 2022). We hypothesize that high-temperature microwave drying has a significant impact on GR and that further research efforts are needed to explore its effects on GR in the treatment of brain diseases.

## 5 Conclusion

The quality markers of GR in the treatment of brain diseases after processing have been preliminarily identified to include gastrodin, S-(4-hydroxybenzyl)-glutathione, and the parishins B, C, and E. These markers may exhibit therapeutic effects against AD, MDD, and CI by acting on the pathways involving *AKT1*, *SRC*, *MAPK*, and other proteins. The processing protocols used can significantly affect the therapeutic components of GR and its effectiveness in treating brain diseases. The therapeutic effects of different processing methods of GR on AD, MDD, and CI were clearly identified. Given the therapeutic purpose of GR, the corresponding optimal processing method can be adopted to improve its efficacy against brain diseases. This strategy is also suggested as an important basis to explain the therapeutic effects of the processing methods of GR against AD, MDD, and CI as well as promoting its clinical applications.

## Data Availability

The original contributions presented in this study are included in the article/Supplementary Material, and any further inquiries may be directed to the corresponding authors.

## Supplementary material

SUPPLEMENT TABLE S1 UPLC-QTOF-MS/MS characterization of the chemical constituents in different processing pieces of *Gastrodia elata* Bl.

## Abbreviations

GR, Gastrodiae rhizoma; AD, Alzheimer’s disease; MDD, major depressive disorder; CI, cerebral ischemia; TCM, traditional Chinese medicine; SD, fresh tubers sliced and dried under the sun; HD, fresh tubers sliced and dried in hot air at 60°C; FD, fresh tubers sliced and freeze-dried; MD, fresh tubers sliced and dried in a microwave; Steam-SD, fresh tubers sliced, steamed, and dried under the sun; Steam-HD, fresh tubers steamed and then dried in hot air at 60°C; Steam-FD, fresh tubers steamed and then dried by freezing; Steam-MD, fresh tubers steamed and then dried in a microwave.
