# Supplementary material for: Identifying the quality markers and optimizing the processing of Gastrodiae rhizoma to treat brain diseases
Source: Front Pharmacol. 2024 Nov 6;15:1396825. doi: 10.3389/fphar.2024.1396825 (PMC11576197; doi:10.3389/fphar.2024.1396825)
Supplement: Supplementary file 1 [file Table5.pdf]

Supplement Tables

Table S5. The F value and P value in multiple comparisons of figure 8

Marker	Groups	Mean Diff.	Type of ANOVA	95.00% CI of diff.	F, DFn, Dfd	P value
Gastrodin	AKT1 vs. MAPK8	1.915	One-Way ANOVA Tukey's post hoc tests	1.493 to 2.337	F (3, 76) = 52.24	<0.0001
	AKT1 vs. SRC	1.475		1.053 to 1.897		<0.0001
	AKT1 vs. EGFR	1.000		0.5777 to 1.422		<0.0001
	MAPK8 vs. SRC	-0.4400		-0.8623 to -0.01774		0.0379
	MAPK8 vs. EGFR	-0.9150		-1.337 to -0.4927		<0.0001
	SRC vs. EGFR	-0.4750		-0.8973 to -0.05274		0.0212
S-(4-hydroxybenzyl)-glutathione	AKT1 vs. MAPK8	1.790	One-Way ANOVA Tukey's post hoc tests	1.589 to 1.991	F (3, 76) = 234.0	<0.0001
	AKT1 vs. SRC	1.420		1.219 to 1.621		<0.0001
	AKT1 vs. EGFR	1.665		1.464 to 1.866		<0.0001
	MAPK8 vs. SRC	-0.3700		-0.5708 to -0.1692		<0.0001
	MAPK8 vs. EGFR	-0.1250		-0.3258 to 0.07583		0.3655
	SRC vs. EGFR	0.2450		0.04417 to 0.4458		0.0104
Parishin C	AKT1 vs. MAPK8	3.070	One-Way ANOVA Tukey's post hoc tests	2.734 to 3.406	F (3, 76) = 196.5	<0.0001
	AKT1 vs. SRC	1.890		1.554 to 2.226		<0.0001
	AKT1 vs. EGFR	1.845		1.509 to 2.181		<0.0001
	MAPK8 vs. SRC	-1.180		-1.516 to -0.8440		<0.0001
	MAPK8 vs. EGFR	-1.225		-1.561 to -0.8890		<0.0001
	SRC vs. EGFR	-0.04500		-0.3810 to 0.2910		0.9850
Glucosyringic acid	AKT1 vs. MAPK8	0.9100	One-Way ANOVA Tukey's post hoc tests	0.5108 to 1.309	F (3, 76) = 19.39	<0.0001
	AKT1 vs. SRC	0.5750		0.1758 to 0.9742		0.0017
	AKT1 vs. EGFR	-0.08000		-0.4792 to 0.3192		0.9524
	MAPK8 vs. SRC	-0.3350		-0.7342 to 0.06419		0.1313
	MAPK8 vs. EGFR	-0.9900		-1.389 to -0.5908		<0.0001
	SRC vs. EGFR	-0.6550		-1.054 to -0.2558		0.0003
