# Supplementary material for: Identifying the quality markers and optimizing the processing of Gastrodiae rhizoma to treat brain diseases
Source: Front Pharmacol. 2024 Nov 6;15:1396825. doi: 10.3389/fphar.2024.1396825 (PMC11576197; doi:10.3389/fphar.2024.1396825)
Supplement: Supplementary file 2 [file Table4.pdf]

Supplement Tables

Table S4. Normality and Lognormality Tests and P value of figure 8

Marker	Groups	P value	Passed normality test (alpha=0.05)	Choice of analytical method
Gastrodin	AKT1	0.0509	Yes	One-Way ANOVA Tukey's post hoc tests
	MAPK8	0.0513	Yes	
	SRC	0.0585	Yes	
	EGFR	0.1798	Yes	
S-(4-hydroxybenzyl)-glutathione	AKT1	0.8594	Yes	One-Way ANOVA Tukey's post hoc tests
	MAPK8	0.8746	Yes	
	SRC	0.2747	Yes	
	EGFR	0.3249	Yes	
Parishin C	AKT1	0.0657	Yes	One-Way ANOVA Tukey's post hoc tests
	MAPK8	0.0567	Yes	
	SRC	0.0845	Yes	
	EGFR	0.0501	Yes	
Glucosyringic acid	AKT1	0.0635	Yes	One-Way ANOVA Tukey's post hoc tests
	MAPK8	0.0522	Yes	
	SRC	0.6689	Yes	
	EGFR	0.5817	Yes	
