# Supplementary material for: Identifying the quality markers and optimizing the processing of Gastrodiae rhizoma to treat brain diseases
Source: Front Pharmacol. 2024 Nov 6;15:1396825. doi: 10.3389/fphar.2024.1396825 (PMC11576197; doi:10.3389/fphar.2024.1396825)
Supplement: Supplementary file 3 [file Table1.pdf]

Supplement Tables

Table S1. UPLC-QTOF-MS/MS Characterization of Chemical Constituents in different processing pieces of *Gastrodia elata* Blume

No.	t_R/min	Identification	formula	$[M-H]^-$ (m/z)	ppm	Fragmentation ions	Ref	class
1	1.72	Galactinol	$C_{12}H_{22}O_{11}$	341.1091	2.05	179.0611 101.0259 89.0267	Massbank	AC
2	2.14	Citric acid	$C_6H_8O_7$	191.0181	-5.76	173.0143 129.0218 111.0117	1	OA
3	3.18	Isomer of Citric acid	$C_6H_8O_7$	191.0219	-6.28	111.0117 87.0079 85.0293	2	OA
4	8.69	Gastrodin	$C_{13}H_{18}O_7$	331.1030	0.30	285.1281 161.0605 123.0572	Std	AC
5	8.98	3-Phenylpropanoic acid	$C_9H_{11}NO_2$	164.0708	-2.44	101.0301 89.0229 85.0331 71.0185 59.0146	Massbank	AC
6	9.48	p-hydroxybenzyl alcohol	$C_7H_8O_2$	123.0436	-8.13	106.9507 88.9437 77.0491	Std	AC
7	10.21	Glucosyringic acid	$C_{15}H_{20}O_{10}$	359.0967	-3.06	341.1210 197.0625 123.0141 95.0163	3	AC
8	10.56	Uracil	$C_4H_4N_2O_2$	111.0181	7.21	101.0239 89.0287 82.6839 78.9586 71.0150	Massbank	NC
9	11.42	Parishin E	$C_{19}H_{24}O_{13}$	459.1152	2.83	459.1531 129.0288 111.0181	Std	AC
10	11.85	s-(4-hydroxybenzyl) -glutathione	$C_{17}H_{23}N_3O_7S$	412.1164	-3.40	306.0810 210.0961 160.0099 143.0518 128.0376 99.0539	2	SC
11	12.00	S-(Gastrodin)-glutathione	$C_{23}H_{33}O_{12}N_3S$	574.1713	1.04	574.1931 306.0845 272.0944 210.0842 179.0502 143.0493 128.0353 99.0579	4	SC
12	12.40	CA(Glu/p-HA-*O*-Met/0)	$C_{20}H_{26}O_{14}$	489.1245	0.20	266.7658 173.0046 111.0082	5	OA
13	14.08	p-hydroxybenz aldehyde	$C_7H_6O_2$	121.0279	-9.09	121.0373	Std	AC
14	15.09	Adenisine	$C_{10}H_{13}N_5O_4$	266.0897	3.01	134.0491 107.0355 92.0266	2	NC

15	15.70	Parishin B	$C_{32}H_{40}O_{19}$	727.2069	-2.34	727.2749 423.1193 369.1395 217.0708 161.0580 111.0160	Std	AC
16	15.92	Parishin V	$C_{38}H_{50}O_{24}$	889.2626	1.35	727.2583 423.1235 397.1448 323.1226 161.0554 111.0181	3	AC
17	16.25	Parishin H	$C_{33}H_{42}O_{20}$	757.2133	-7.66	485.0511 459.1487 385.1067 336.5907 256.0884 190.9654 183.8740 175.0481 111.0160	3	AC
18	16.54	Parishin C	$C_{32}H_{40}O_{19}$	727.2069	-2.34	727.2749 441.1478 423.1361 397.1366 217.0678 161.0580	Std	AC
19	17.10	Parishin M	$C_{33}H_{42}O_{20}$	757.2242	6.74	626.0646 161.0554 111.0138	3	AC
20	17.52	Parishin K	$C_{33}H_{42}O_{19}$	741.2269	3.64	473.1661 169.0247 123.0550 83.0193	3	AC
21	17.70	Parishin F	$C_{51}H_{66}O_{30}$	1157.3412	-12.87	889.3603 757.2749 585.2158 423.1361 161.0501 112.9943	3	AC
22	18.60	4,4'-Dihydroxybenzyl sulfoxide	$C_{14}H_{14}SO_3$	261.0565	-7.66	111.0117 81.7649	3，6	SC
23	18.88	Parishin A	$C_{45}H_{56}O_{25}$	995.3074	4.22	995.4046 727.2804 441.1435 423.1361 397.1570 217.0678 161.0580 111.0160	Std	AC
24	19.20	Parishin L	$C_{46}H_{58}O_{26}$	1025.3212	7.22	727.2252 423.1066 161.0528 111.0095	4	AC
25	19.71	Parishin W	$C_{26}H_{30}O_{14}$	565.1519	-6.72	305.0824 111.0117	3	AC
26	20.12	Gastrodioside	$C_{20}H_{24}O_8$	391.1350	-10.99	22.0911 161.0424 123.0504 121.0328 93.0370	3,6	AC
27	21.20	Azelaic acid	$C_9H_{16}O_4$	187.0973	1.60	187.1003 125.0984 116.9348	Massbank	OA
28	21.52	Parishin R	$C_{52}H_{62}O_{26}$	1101.3513	5.63	727.1869 346.1662 161.0400	5	AC
29	23.51	CA(CA/0/p-HA-*Rha*/0)/p-HA-p-HPP/0)	$C_{41}H_{46}O_{20}$	857.2548	5.13	423.1031 362.0992 111.0091	5	OA
30	23.98	4,4’-dihydroxydibenzyl ether	$C_{14}H_{14}O_3$	229.0873	3.49	121.0306 107.0524 93.0370	2	AC
31	24.08	Parishin D	$C_{20}H_{20}O_9$	403.1042	3.22	191.0276 111.0095 87.0118	6	AC
32	26.36	Grossamide	$C_{36}H_{36}N_2O_8$	623.2384	-1.44	460.2022 297.1312	3	NC

AC: aromatic compounds; NC: nitrogen compounds; SC: sulfur compounds; QA: organic acids

References:

[1] Lai, C. J., Yuan, Y., Liu, D. H., Kang, C. Z., Zhang, Y., Zha, L., Kang, L., Chen, T., Nan, T. G., Hao, Q. X., and Huang, L. Q. (2017). Untargeted metabolite analysis-based UHPLC-Q-TOF-MS reveals significant enrichment of p-hydroxybenzyl dimers of citric acids in fresh beige-scape *Gastrodia elata* (Wutianma). J. Pharm. Biomed. Anal. 140, 287-294. doi: 10.1016/j.jpba.2017.03.055

[2] Yun, L., Zhi, W. W., Yan, L. G., Hong, L. Z., Da, H. L., and Xiao, W. (2016). Study on the determination of chemical constituents in Rhizoma gastrodiae and Rhizoma erythrofolia by HPLC-ESI-TOF/MS. J. Research and development of natural products. 28(11), 1758-1763.

[3] Li, Z., Wang, Y., Ouyang, H., Lu, Y., Qiu, Y., Feng, Y., Jiang, H., Zhou, X., and Yang, S. (2015). A novel dereplication strategy for the identification of two new trace compounds in the extract of *Gastrodia elata* using UHPLC/Q-TOF-MS/MS. J. Chromatogr. B. 988, 45-52. doi: 10.1016/j.jchromb.2015.02.020

[4] Tang, C., Wang, L., Li, J., Liu, X., Cheng, M., and Xiao, H. (2015). Analysis of the metabolic profile of parishin by ultra-performance liquid chromatography/quadrupole-time of flight mass spectrometry. Biomed. Chromatogr. 29(12), 1913-20. doi: 10.1002/bmc.3516

[5] Tang, C., Wang, L., Liu, X., Cheng, M., and Xiao, H. (2016). Chemical fingerprint and metabolic profile analysis of ethyl acetate fraction of *Gastrodia elata* by ultra performance liquid chromatography/quadrupole-time of flight mass spectrometry. J. Chromatogr. B. 1011, 233-9. doi: 10.1016/j.jchromb.2015.09.043

[6] Ye, X., Wang, Y., Zhao, J., Wang, M., Avula, B., Peng, Q., Ouyang, H., Lingyun, Z., Zhang, J., and Khan, I. A. (2019). Identification and Characterization of Key Chemical Constituents in Processed *Gastrodia elata* Using UHPLC-MS/MS and Chemometric Methods. J. Anal. Methods Chem. 2019, 4396201. doi: 10.1155/2019/439620
