# Supplementary material for: Identifying the quality markers and optimizing the processing of Gastrodiae rhizoma to treat brain diseases
Source: Front Pharmacol. 2024 Nov 6;15:1396825. doi: 10.3389/fphar.2024.1396825 (PMC11576197; doi:10.3389/fphar.2024.1396825)
Supplement: Supplementary file 4 [file Table2.pdf]

Supplement Tables

Table S2. Normality and Lognormality Tests and P value of figure 3

Marker	Groups	P value	Passed normality test (alpha=0.05)	Choice of analytical method
Galactinol	No steam-Sun drying	0.5520	Yes	One-Way ANOVA Tukey's post hoc tests
	No steam-Hot air drying	0.9374	Yes	
	No steam-Freeze drying	0.1322	Yes	
	No steam-Microwave drying	0.9181	Yes	
	Steam-Sun drying	0.3916	Yes	
	Steam-Hot air drying	0.0567	Yes	
	Steam-Freeze drying	0.6993	Yes	
	Steam-Microwave drying	0.9245	Yes	
Citric acid	No steam-Sun drying	0.4763	Yes	One-Way ANOVA Tukey's post hoc tests
	No steam-Hot air drying	0.7500	Yes	
	No steam-Freeze drying	0.5658	Yes	
	No steam-Microwave drying	0.3477	Yes	
	Steam-Sun drying	0.8859	Yes	
	Steam-Hot air drying	0.9343	Yes	
	Steam-Freeze drying	0.1224	Yes	
	Steam-Microwave drying	0.8693	Yes	
Gastrodin	No steam-Sun drying	0.7500	Yes	One-Way ANOVA Tukey's post hoc tests
	No steam-Hot air drying	0.7500	Yes	
	No steam-Freeze drying	0.8248	Yes	
	No steam-Microwave drying	0.3972	Yes	
	Steam-Sun drying	0.0560	Yes	
	Steam-Hot air drying	0.2426	Yes	
	Steam-Freeze drying	0.1199	Yes	
	Steam-Microwave drying	0.6701	Yes	
Glucosyringic acid	No steam-Sun drying	0.1985	Yes	One-Way ANOVA Tukey's post hoc tests
	No steam-Hot air drying	0.7500	Yes	
	No steam-Freeze drying	0.2536	Yes	
	No steam-Microwave drying	0.0556	Yes	
	Steam-Sun drying	0.3487	Yes	
	Steam-Hot air drying	0.6524	Yes	
	Steam-Freeze drying	0.6916	Yes	
	Steam-Microwave drying	0.4663	Yes	
Uracil	No steam-Sun drying	0.0717	Yes	One-Way ANOVA Tukey's post hoc tests
	No steam-Hot air drying	0.7500	Yes	
	No steam-Freeze drying	0.2055	Yes	
	No steam-Microwave drying	0.7503	Yes	
	Steam-Sun drying	0.0578	Yes	
	Steam-Hot air drying	0.8412	Yes	
	Steam-Freeze drying	0.0563	Yes	
	Steam-Microwave drying	0.5160	Yes	
Parishin E	No steam-Sun drying	0.6523	Yes	One-Way ANOVA Tukey's post hoc tests
	No steam-Hot air drying	0.6523	Yes	

	No steam-Freeze drying	0.7500	Yes	
	No steam-Microwave drying	0.0599	Yes	
	Steam-Sun drying	0.7187	Yes	
	Steam-Hot air drying	0.8131	Yes	
	Steam-Freeze drying	0.6933	Yes	
	Steam-Microwave drying	0.8895	Yes	
S-(4-hydroxybenzyl)-glutathione	No steam-Sun drying	0.8266	Yes	One-Way ANOVA Tukey's post hoc tests
	No steam-Hot air drying	0.7500	Yes	
	No steam-Freeze drying	0.0529	Yes	
	No steam-Microwave drying	0.4719	Yes	
	Steam-Sun drying	0.2366	Yes	
	Steam-Hot air drying	0.9542	Yes	
	Steam-Freeze drying	0.3202	Yes	
	Steam-Microwave drying	0.4100	Yes	
Parishin B	No steam-Sun drying	0.4166	Yes	One-Way ANOVA Tukey's post hoc tests
	No steam-Hot air drying	0.7500	Yes	
	No steam-Freeze drying	0.8696	Yes	
	No steam-Microwave drying	0.3327	Yes	
	Steam-Sun drying	0.667	Yes	
	Steam-Hot air drying	0.7354	Yes	
	Steam-Freeze drying	0.1340	Yes	
	Steam-Microwave drying	0.4325	Yes	
Parishin C	No steam-Sun drying	0.2088	Yes	One-Way ANOVA Tukey's post hoc tests
	No steam-Hot air drying	0.7500	Yes	
	No steam-Freeze drying	0.2207	Yes	
	No steam-Microwave drying	0.6594	Yes	
	Steam-Sun drying	0.6191	Yes	
	Steam-Hot air drying	0.9551	Yes	
	Steam-Freeze drying	0.2108	Yes	
	Steam-Microwave drying	0.4607	Yes	
Parishin A	No steam-Sun drying	0.3608	Yes	One-Way ANOVA Tukey's post hoc tests
	No steam-Hot air drying	0.7500	Yes	
	No steam-Freeze drying	0.5436	Yes	
	No steam-Microwave drying	0.4245	Yes	
	Steam-Sun drying	0.2924	Yes	
	Steam-Hot air drying	0.1413	Yes	
	Steam-Freeze drying	0.525	Yes	
	Steam-Microwave drying	0.0476	Yes	
Parishin D	No steam-Sun drying	0.3127	Yes	One-Way ANOVA Tukey's post hoc tests
	No steam-Hot air drying	0.7500	Yes	
	No steam-Freeze drying	0.0906	Yes	
	No steam-Microwave drying	0.5181	Yes	
	Steam-Sun drying	0.2624	Yes	
	Steam-Hot air drying	0.8894	Yes	
	Steam-Freeze drying	0.1245	Yes	
	Steam-Microwave drying	0.5580	Yes	
Parishin L	No steam-Sun drying	0.9653	Yes	One-Way ANOVA Tukey's post hoc tests
	No steam-Hot air drying	0.7500	Yes	

	No steam-Freeze drying	0.0659	Yes	
	No steam-Microwave drying	0.9331	Yes	
	Steam-Sun drying	0.6165	Yes	
	Steam-Hot air drying	0.8073	Yes	
	Steam-Freeze drying	0.4061	Yes	
	Steam-Microwave drying	0.1855	Yes	
Parishin R	No steam-Sun drying	0.0847	Yes	One-Way ANOVA Tukey’s post hoc tests
	No steam-Hot air drying	0.7500	Yes	
	No steam-Freeze drying	0.4192	Yes	
	No steam-Microwave drying	0.1928	Yes	
	Steam-Sun drying	0.8526	Yes	
	Steam-Hot air drying	0.0580	Yes	
	Steam-Freeze drying	0.5038	Yes	
	Steam-Microwave drying	0.7694	Yes	
