# Supplementary material for: Identifying the quality markers and optimizing the processing of Gastrodiae rhizoma to treat brain diseases
Source: Front Pharmacol. 2024 Nov 6;15:1396825. doi: 10.3389/fphar.2024.1396825 (PMC11576197; doi:10.3389/fphar.2024.1396825)
Supplement: Supplementary file 5 [file Table3.pdf]

Supplement Tables

Table S3. The F value and P value in multiple comparisons of figure 3

Marker	Groups	Mean Diff.	Type of ANOVA	F, DFn, Dfd	P Value
Galactinol	No steam-Sun drying vs. No steam-Hot air drying	-3.312	One-Way ANOVA Tukey's post hoc tests	F (7, 16) = 1.660	0.9818
	No steam-Sun drying vs. No steam-Freeze drying	-0.7721			>0.9999
	No steam-Sun drying vs. No steam-Microwave drying	0.07840			>0.9999
	No steam-Sun drying vs. Steam-Sun drying	-2.571			0.9958
	No steam-Sun drying vs. Steam-Hot air drying	7.071			0.5568
	No steam-Sun drying vs. Steam-Freeze drying	-3.372			0.9799
	No steam-Sun drying vs. Steam-Microwave drying	0.1865			>0.9999
	No steam-Hot air drying vs. No steam-Freeze drying	2.540			0.9961
	No steam-Hot air drying vs. No steam-Microwave drying	3.391			0.9792
	No steam-Hot air drying vs. Steam-Sun drying	0.7409			>0.9999
	No steam-Hot air drying vs. Steam-Hot air drying	10.38			0.1571
	No steam-Hot air drying vs. Steam-Freeze drying	-0.05945			>0.9999
	No steam-Hot air drying vs. Steam-Microwave drying	3.499			0.9754
	No steam-Freeze drying vs. No steam-Microwave drying	0.8505			>0.9999
	No steam-Freeze drying vs. Steam-Sun drying	-1.799			0.9996
	No steam-Freeze drying vs. Steam-Hot air drying	7.843			0.4370
	No steam-Freeze drying vs. Steam-Freeze drying	-2.600			0.9955
	No steam-Freeze drying vs. Steam-Microwave drying	0.9587			>0.9999
	No steam-Microwave drying vs. Steam-Sun drying	-2.650			0.9950
	No steam-Microwave drying vs. Steam-Hot air drying	6.992			0.5694
	No steam-Microwave drying vs. Steam-Freeze drying	-3.450			0.9772
	No steam-Microwave drying vs. Steam-Microwave drying	0.1081			>0.9999
	Steam-Sun drying vs. Steam-Hot air drying	9.642			0.2180
	Steam-Sun drying vs. Steam-Freeze drying	-0.8003			>0.9999
	Steam-Sun drying vs. Steam-Microwave drying	2.758			0.9936
	Steam-Hot air drying vs. Steam-Freeze drying	-10.44			0.1529
	Steam-Hot air drying vs. Steam-Microwave drying	-6.884			0.5868
	Steam-Freeze drying vs. Steam-Microwave drying	3.558			0.9731
Citric acid	No steam-Sun drying vs. No steam-Hot air drying	-7.121	One-Way ANOVA Tukey's post hoc tests	F (7, 16) = 53.02	0.0003
	No steam-Sun drying vs. No steam-Freeze drying	0.2581			>0.9999
	No steam-Sun drying vs. No steam-Microwave drying	10.09			<0.0001
	No steam-Sun drying vs. Steam-Sun drying	10.97			<0.0001
	No steam-Sun drying vs. Steam-Hot air drying	1.552			0.8742
	No steam-Sun drying vs. Steam-Freeze drying	6.317			0.0012
	No steam-Sun drying vs. Steam-Microwave drying	5.952			0.0021
	No steam-Hot air drying vs. No steam-Freeze drying	7.379			0.0002
	No steam-Hot air drying vs. No steam-Microwave drying	17.21			<0.0001
	No steam-Hot air drying vs. Steam-Sun drying	18.09			<0.0001
	No steam-Hot air drying vs. Steam-Hot air drying	8.673			<0.0001
	No steam-Hot air drying vs. Steam-Freeze drying	13.44			<0.0001
	No steam-Hot air drying vs. Steam-Microwave drying	13.07			<0.0001

	No steam-Freeze drying vs. No steam-Microwave drying	9.832			<0.0001
	No steam-Freeze drying vs. Steam-Sun drying	10.71			<0.0001
	No steam-Freeze drying vs. Steam-Hot air drying	1.294			0.9454
	No steam-Freeze drying vs. Steam-Freeze drying	6.059			0.0018
	No steam-Freeze drying vs. Steam-Microwave drying	5.694			0.0032
	No steam-Microwave drying vs. Steam-Sun drying	0.8789			0.9934
	No steam-Microwave drying vs. Steam-Hot air drying	-8.538			<0.0001
	No steam-Microwave drying vs. Steam-Freeze drying	-3.773			0.0762
	No steam-Microwave drying vs. Steam-Microwave drying	-4.138			0.0426
	Steam-Sun drying vs. Steam-Hot air drying	-9.417			<0.0001
	Steam-Sun drying vs. Steam-Freeze drying	-4.651			0.0183
	Steam-Sun drying vs. Steam-Microwave drying	-5.017			0.0100
	Steam-Hot air drying vs. Steam-Freeze drying	4.765			0.0152
	Steam-Hot air drying vs. Steam-Microwave drying	4.400			0.0278
	Steam-Freeze drying vs. Steam-Microwave drying	-0.3652			>0.9999
Gastrodin	No steam-Sun drying vs. No steam-Hot air drying	-0.09135	One-Way ANOVA Tukey’s post hoc tests	F (7, 16) = 196.4	>0.9999
	No steam-Sun drying vs. No steam-Freeze drying	-0.1319			>0.9999
	No steam-Sun drying vs. No steam-Microwave drying	-7.405			<0.0001
	No steam-Sun drying vs. Steam-Sun drying	-5.135			<0.0001
	No steam-Sun drying vs. Steam-Hot air drying	-4.068			<0.0001
	No steam-Sun drying vs. Steam-Freeze drying	-6.772			<0.0001
	No steam-Sun drying vs. Steam-Microwave drying	-8.908			<0.0001
	No steam-Hot air drying vs. No steam-Freeze drying	-0.04050			>0.9999
	No steam-Hot air drying vs. No steam-Microwave drying	-7.313			<0.0001
	No steam-Hot air drying vs. Steam-Sun drying	-5.044			<0.0001
	No steam-Hot air drying vs. Steam-Hot air drying	-3.976			<0.0001
	No steam-Hot air drying vs. Steam-Freeze drying	-6.680			<0.0001
	No steam-Hot air drying vs. Steam-Microwave drying	-8.817			<0.0001
	No steam-Freeze drying vs. No steam-Microwave drying	-7.273			<0.0001
	No steam-Freeze drying vs. Steam-Sun drying	-5.004			<0.0001
	No steam-Freeze drying vs. Steam-Hot air drying	-3.936			<0.0001
	No steam-Freeze drying vs. Steam-Freeze drying	-6.640			<0.0001
	No steam-Freeze drying vs. Steam-Microwave drying	-8.776			<0.0001
	No steam-Microwave drying vs. Steam-Sun drying	2.269			0.0002
	No steam-Microwave drying vs. Steam-Hot air drying	3.337			<0.0001
	No steam-Microwave drying vs. Steam-Freeze drying	0.6329			0.6637
	No steam-Microwave drying vs. Steam-Microwave drying	-1.504			0.0137
	Steam-Sun drying vs. Steam-Hot air drying	1.068			0.1292
	Steam-Sun drying vs. Steam-Freeze drying	-1.636			0.0067
	Steam-Sun drying vs. Steam-Microwave drying	-3.773			<0.0001
	Steam-Hot air drying vs. Steam-Freeze drying	-2.704			<0.0001
	Steam-Hot air drying vs. Steam-Microwave drying	-4.840			<0.0001
	Steam-Freeze drying vs. Steam-Microwave drying	-2.136			0.0005
Glucosyringic acid	No steam-Sun drying vs. No steam-Hot air drying	8.843	One-Way ANOVA Tukey’s post hoc tests	F (7, 16) = 255.3	<0.0001
	No steam-Sun drying vs. No steam-Freeze drying	2.219			0.1104
	No steam-Sun drying vs. No steam-Microwave drying	19.42			<0.0001
	No steam-Sun drying vs. Steam-Sun drying	19.32			<0.0001
	No steam-Sun drying vs. Steam-Hot air drying	20.60			<0.0001

	No steam-Sun drying vs. Steam-Freeze drying	17.97			<0.0001
	No steam-Sun drying vs. Steam-Microwave drying	16.78			<0.0001
	No steam-Hot air drying vs. No steam-Freeze drying	-6.624			<0.0001
	No steam-Hot air drying vs. No steam-Microwave drying	10.57			<0.0001
	No steam-Hot air drying vs. Steam-Sun drying	10.48			<0.0001
	No steam-Hot air drying vs. Steam-Hot air drying	11.76			<0.0001
	No steam-Hot air drying vs. Steam-Freeze drying	9.123			<0.0001
	No steam-Hot air drying vs. Steam-Microwave drying	7.939			<0.0001
	No steam-Freeze drying vs. No steam-Microwave drying	17.20			<0.0001
	No steam-Freeze drying vs. Steam-Sun drying	17.10			<0.0001
	No steam-Freeze drying vs. Steam-Hot air drying	18.38			<0.0001
	No steam-Freeze drying vs. Steam-Freeze drying	15.75			<0.0001
	No steam-Freeze drying vs. Steam-Microwave drying	14.56			<0.0001
	No steam-Microwave drying vs. Steam-Sun drying	-0.09448			>0.9999
	No steam-Microwave drying vs. Steam-Hot air drying	1.183			0.7362
	No steam-Microwave drying vs. Steam-Freeze drying	-1.451			0.5229
	No steam-Microwave drying vs. Steam-Microwave drying	-2.634			0.0391
	Steam-Sun drying vs. Steam-Hot air drying	1.278			0.6623
	Steam-Sun drying vs. Steam-Freeze drying	-1.356			0.5990
	Steam-Sun drying vs. Steam-Microwave drying	-2.540			0.0498
	Steam-Hot air drying vs. Steam-Freeze drying	-2.634			0.0391
	Steam-Hot air drying vs. Steam-Microwave drying	-3.817			0.0017
	Steam-Freeze drying vs. Steam-Microwave drying	-1.183			0.7363
Uracil	No steam-Sun drying vs. No steam-Hot air drying	0.08057	One-Way ANOVA Tukey’s post hoc tests	F (7, 16) = 4.272	>0.9999
	No steam-Sun drying vs. No steam-Freeze drying	-1.599			0.6298
	No steam-Sun drying vs. No steam-Microwave drying	-3.504			0.0200
	No steam-Sun drying vs. Steam-Sun drying	-3.122			0.0456
	No steam-Sun drying vs. Steam-Hot air drying	-2.323			0.2204
	No steam-Sun drying vs. Steam-Freeze drying	-2.014			0.3669
	No steam-Sun drying vs. Steam-Microwave drying	-1.971			0.3915
	No steam-Hot air drying vs. No steam-Freeze drying	-1.679			0.5759
	No steam-Hot air drying vs. No steam-Microwave drying	-3.585			0.0168
	No steam-Hot air drying vs. Steam-Sun drying	-3.202			0.0384
	No steam-Hot air drying vs. Steam-Hot air drying	-2.404			0.1907
	No steam-Hot air drying vs. Steam-Freeze drying	-2.095			0.3237
	No steam-Hot air drying vs. Steam-Microwave drying	-2.051			0.3465
	No steam-Freeze drying vs. No steam-Microwave drying	-1.906			0.4301
	No steam-Freeze drying vs. Steam-Sun drying	-1.523			0.6799
	No steam-Freeze drying vs. Steam-Hot air drying	-0.7245			0.9896
	No steam-Freeze drying vs. Steam-Freeze drying	-0.4155			0.9997
	No steam-Freeze drying vs. Steam-Microwave drying	-0.3722			0.9998
	No steam-Microwave drying vs. Steam-Sun drying	0.3828			0.9998
	No steam-Microwave drying vs. Steam-Hot air drying	1.181			0.8749
	No steam-Microwave drying vs. Steam-Freeze drying	1.490			0.7012
	No steam-Microwave drying vs. Steam-Microwave drying	1.534			0.6729
	Steam-Sun drying vs. Steam-Hot air drying	0.7985			0.9820
	Steam-Sun drying vs. Steam-Freeze drying	1.107			0.9057
	Steam-Sun drying vs. Steam-Microwave drying	1.151			0.8882

	Steam-Hot air drying vs. Steam-Freeze drying	0.3090			>0.9999
	Steam-Hot air drying vs. Steam-Microwave drying	0.3523			0.9999
	Steam-Freeze drying vs. Steam-Microwave drying	0.04330			>0.9999
Parishin E	No steam-Sun drying vs. No steam-Hot air drying	-0.3342	One-Way ANOVA Tukey's post hoc tests	F (7, 16) = 37.07	>0.9999
	No steam-Sun drying vs. No steam-Freeze drying	-11.23			0.0064
	No steam-Sun drying vs. No steam-Microwave drying	-16.44			0.0001
	No steam-Sun drying vs. Steam-Sun drying	-21.10			<0.0001
	No steam-Sun drying vs. Steam-Hot air drying	-27.30			<0.0001
	No steam-Sun drying vs. Steam-Freeze drying	-20.06			<0.0001
	No steam-Sun drying vs. Steam-Microwave drying	-26.43			<0.0001
	No steam-Hot air drying vs. No steam-Freeze drying	-10.90			0.0084
	No steam-Hot air drying vs. No steam-Microwave drying	-16.10			0.0002
	No steam-Hot air drying vs. Steam-Sun drying	-20.77			<0.0001
	No steam-Hot air drying vs. Steam-Hot air drying	-26.97			<0.0001
	No steam-Hot air drying vs. Steam-Freeze drying	-19.73			<0.0001
	No steam-Hot air drying vs. Steam-Microwave drying	-26.09			<0.0001
	No steam-Freeze drying vs. No steam-Microwave drying	-5.204			0.4565
	No steam-Freeze drying vs. Steam-Sun drying	-9.867			0.0188
	No steam-Freeze drying vs. Steam-Hot air drying	-16.07			0.0002
	No steam-Freeze drying vs. Steam-Freeze drying	-8.827			0.0420
	No steam-Freeze drying vs. Steam-Microwave drying	-15.19			0.0003
	No steam-Microwave drying vs. Steam-Sun drying	-4.663			0.5825
	No steam-Microwave drying vs. Steam-Hot air drying	-10.86			0.0086
	No steam-Microwave drying vs. Steam-Freeze drying	-3.624			0.8171
	No steam-Microwave drying vs. Steam-Microwave drying	-9.990			0.0171
	Steam-Sun drying vs. Steam-Hot air drying	-6.198			0.2639
	Steam-Sun drying vs. Steam-Freeze drying	1.039			0.9998
	Steam-Sun drying vs. Steam-Microwave drying	-5.327			0.4293
	Steam-Hot air drying vs. Steam-Freeze drying	7.238			0.1341
	Steam-Hot air drying vs. Steam-Microwave drying	0.8713			>0.9999
	Steam-Freeze drying vs. Steam-Microwave drying	-6.366			0.2380
S-(4-hydroxybenzyl)-glutathione	No steam-Sun drying vs. No steam-Hot air drying	0.2379	One-Way ANOVA Tukey's post hoc tests	F (7, 16) = 169.0	>0.9999
	No steam-Sun drying vs. No steam-Freeze drying	1.284			0.8273
	No steam-Sun drying vs. No steam-Microwave drying	-14.91			<0.0001
	No steam-Sun drying vs. Steam-Sun drying	-11.77			<0.0001
	No steam-Sun drying vs. Steam-Hot air drying	0.4463			0.9995
	No steam-Sun drying vs. Steam-Freeze drying	-14.96			<0.0001
	No steam-Sun drying vs. Steam-Microwave drying	-16.89			<0.0001
	No steam-Hot air drying vs. No steam-Freeze drying	1.046			0.9290
	No steam-Hot air drying vs. No steam-Microwave drying	-15.15			<0.0001
	No steam-Hot air drying vs. Steam-Sun drying	-12.01			<0.0001
	No steam-Hot air drying vs. Steam-Hot air drying	0.2084			>0.9999
	No steam-Hot air drying vs. Steam-Freeze drying	-15.20			<0.0001
	No steam-Hot air drying vs. Steam-Microwave drying	-17.13			<0.0001
	No steam-Freeze drying vs. No steam-Microwave drying	-16.20			<0.0001
	No steam-Freeze drying vs. Steam-Sun drying	-13.05			<0.0001
	No steam-Freeze drying vs. Steam-Hot air drying	-0.8381			0.9771
	No steam-Freeze drying vs. Steam-Freeze drying	-16.25			<0.0001

	No steam-Freeze drying vs. Steam-Microwave drying	-18.17			<0.0001
	No steam-Microwave drying vs. Steam-Sun drying	3.141			0.0451
	No steam-Microwave drying vs. Steam-Hot air drying	15.36			<0.0001
	No steam-Microwave drying vs. Steam-Freeze drying	-0.05196			>0.9999
	No steam-Microwave drying vs. Steam-Microwave drying	-1.977			0.3932
	Steam-Sun drying vs. Steam-Hot air drying	12.22			<0.0001
	Steam-Sun drying vs. Steam-Freeze drying	-3.193			0.0404
	Steam-Sun drying vs. Steam-Microwave drying	-5.119			0.0006
	Steam-Hot air drying vs. Steam-Freeze drying	-15.41			<0.0001
	Steam-Hot air drying vs. Steam-Microwave drying	-17.33			<0.0001
	Steam-Freeze drying vs. Steam-Microwave drying	-1.925			0.4238
Parishin B	No steam-Sun drying vs. No steam-Hot air drying	3.353	One-Way ANOVA Tukey's post hoc tests	F (7, 16) = 65.89	0.8852
	No steam-Sun drying vs. No steam-Freeze drying	-4.807			0.5889
	No steam-Sun drying vs. No steam-Microwave drying	-27.74			<0.0001
	No steam-Sun drying vs. Steam-Sun drying	-28.74			<0.0001
	No steam-Sun drying vs. Steam-Hot air drying	-27.72			<0.0001
	No steam-Sun drying vs. Steam-Freeze drying	-28.20			<0.0001
	No steam-Sun drying vs. Steam-Microwave drying	-30.89			<0.0001
	No steam-Hot air drying vs. No steam-Freeze drying	-8.161			0.0856
	No steam-Hot air drying vs. No steam-Microwave drying	-31.10			<0.0001
	No steam-Hot air drying vs. Steam-Sun drying	-32.09			<0.0001
	No steam-Hot air drying vs. Steam-Hot air drying	-31.07			<0.0001
	No steam-Hot air drying vs. Steam-Freeze drying	-31.55			<0.0001
	No steam-Hot air drying vs. Steam-Microwave drying	-34.24			<0.0001
	No steam-Freeze drying vs. No steam-Microwave drying	-22.94			<0.0001
	No steam-Freeze drying vs. Steam-Sun drying	-23.93			<0.0001
	No steam-Freeze drying vs. Steam-Hot air drying	-22.91			<0.0001
	No steam-Freeze drying vs. Steam-Freeze drying	-23.39			<0.0001
	No steam-Freeze drying vs. Steam-Microwave drying	-26.08			<0.0001
	No steam-Microwave drying vs. Steam-Sun drying	-0.9928			>0.9999
	No steam-Microwave drying vs. Steam-Hot air drying	0.02570			>0.9999
	No steam-Microwave drying vs. Steam-Freeze drying	-0.4561			>0.9999
	No steam-Microwave drying vs. Steam-Microwave drying	-3.142			0.9141
	Steam-Sun drying vs. Steam-Hot air drying	1.019			0.9999
	Steam-Sun drying vs. Steam-Freeze drying	0.5368			>0.9999
	Steam-Sun drying vs. Steam-Microwave drying	-2.150			0.9880
	Steam-Hot air drying vs. Steam-Freeze drying	-0.4818			>0.9999
	Steam-Hot air drying vs. Steam-Microwave drying	-3.168			0.9108
	Steam-Freeze drying vs. Steam-Microwave drying	-2.686			0.9599
Parishin C	No steam-Sun drying vs. No steam-Hot air drying	0.1356	One-Way ANOVA Tukey's post hoc tests	F (7, 16) = 62.40	>0.9999
	No steam-Sun drying vs. No steam-Freeze drying	-1.244			0.8807
	No steam-Sun drying vs. No steam-Microwave drying	-9.426			<0.0001
	No steam-Sun drying vs. Steam-Sun drying	-9.154			<0.0001
	No steam-Sun drying vs. Steam-Hot air drying	-9.617			<0.0001
	No steam-Sun drying vs. Steam-Freeze drying	-9.952			<0.0001
	No steam-Sun drying vs. Steam-Microwave drying	-13.16			<0.0001
	No steam-Hot air drying vs. No steam-Freeze drying	-1.379			0.8180
	No steam-Hot air drying vs. No steam-Microwave drying	-9.562			<0.0001

	No steam-Hot air drying vs. Steam-Sun drying	-9.290			<0.0001
	No steam-Hot air drying vs. Steam-Hot air drying	-9.753			<0.0001
	No steam-Hot air drying vs. Steam-Freeze drying	-10.09			<0.0001
	No steam-Hot air drying vs. Steam-Microwave drying	-13.30			<0.0001
	No steam-Freeze drying vs. No steam-Microwave drying	-8.183			<0.0001
	No steam-Freeze drying vs. Steam-Sun drying	-7.911			<0.0001
	No steam-Freeze drying vs. Steam-Hot air drying	-8.373			<0.0001
	No steam-Freeze drying vs. Steam-Freeze drying	-8.708			<0.0001
	No steam-Freeze drying vs. Steam-Microwave drying	-11.92			<0.0001
	No steam-Microwave drying vs. Steam-Sun drying	0.2719			>0.9999
	No steam-Microwave drying vs. Steam-Hot air drying	-0.1908			>0.9999
	No steam-Microwave drying vs. Steam-Freeze drying	-0.5256			0.9990
	No steam-Microwave drying vs. Steam-Microwave drying	-3.737			0.0197
	Steam-Sun drying vs. Steam-Hot air drying	-0.4627			0.9996
	Steam-Sun drying vs. Steam-Freeze drying	-0.7974			0.9874
	Steam-Sun drying vs. Steam-Microwave drying	-4.009			0.0113
	Steam-Hot air drying vs. Steam-Freeze drying	-0.3348			>0.9999
	Steam-Hot air drying vs. Steam-Microwave drying	-3.547			0.0291
	Steam-Freeze drying vs. Steam-Microwave drying	-3.212			0.0567
Parishin A	No steam-Sun drying vs. No steam-Hot air drying	10.76	One-Way ANOVA Tukey's post hoc tests	F (7, 16) = 113.0	0.0232
	No steam-Sun drying vs. No steam-Freeze drying	-7.732			0.1694
	No steam-Sun drying vs. No steam-Microwave drying	-33.46			<0.0001
	No steam-Sun drying vs. Steam-Sun drying	-37.77			<0.0001
	No steam-Sun drying vs. Steam-Hot air drying	-37.09			<0.0001
	No steam-Sun drying vs. Steam-Freeze drying	-41.98			<0.0001
	No steam-Sun drying vs. Steam-Microwave drying	-39.73			<0.0001
	No steam-Hot air drying vs. No steam-Freeze drying	-18.50			0.0001
	No steam-Hot air drying vs. No steam-Microwave drying	-44.22			<0.0001
	No steam-Hot air drying vs. Steam-Sun drying	-48.54			<0.0001
	No steam-Hot air drying vs. Steam-Hot air drying	-47.85			<0.0001
	No steam-Hot air drying vs. Steam-Freeze drying	-52.74			<0.0001
	No steam-Hot air drying vs. Steam-Microwave drying	-50.50			<0.0001
	No steam-Freeze drying vs. No steam-Microwave drying	-25.72			<0.0001
	No steam-Freeze drying vs. Steam-Sun drying	-30.04			<0.0001
	No steam-Freeze drying vs. Steam-Hot air drying	-29.36			<0.0001
	No steam-Freeze drying vs. Steam-Freeze drying	-34.25			<0.0001
	No steam-Freeze drying vs. Steam-Microwave drying	-32.00			<0.0001
	No steam-Microwave drying vs. Steam-Sun drying	-4.317			0.7711
	No steam-Microwave drying vs. Steam-Hot air drying	-3.632			0.8846
	No steam-Microwave drying vs. Steam-Freeze drying	-8.524			0.1040
	No steam-Microwave drying vs. Steam-Microwave drying	-6.279			0.3725
	Steam-Sun drying vs. Steam-Hot air drying	0.6850			>0.9999
	Steam-Sun drying vs. Steam-Freeze drying	-4.207			0.7917
	Steam-Sun drying vs. Steam-Microwave drying	-1.961			0.9956
	Steam-Hot air drying vs. Steam-Freeze drying	-4.892			0.6545
	Steam-Hot air drying vs. Steam-Microwave drying	-2.646			0.9755
	Steam-Freeze drying vs. Steam-Microwave drying	2.245			0.9902
Parishin D	No steam-Sun drying vs. No steam-Hot air drying	0.05678	One-Way ANOVA	F (7, 16) = 35.79	>0.9999

	No steam-Sun drying vs. No steam-Freeze drying	-7.809	Tukey's post hoc tests		<0.0001
	No steam-Sun drying vs. No steam-Microwave drying	-0.5255			0.9865
	No steam-Sun drying vs. Steam-Sun drying	-0.7378			0.9212
	No steam-Sun drying vs. Steam-Hot air drying	-0.7047			0.9366
	No steam-Sun drying vs. Steam-Freeze drying	-0.8756			0.8350
	No steam-Sun drying vs. Steam-Microwave drying	-0.7643			0.9074
	No steam-Hot air drying vs. No steam-Freeze drying	-7.866			<0.0001
	No steam-Hot air drying vs. No steam-Microwave drying	-0.5823			0.9761
	No steam-Hot air drying vs. Steam-Sun drying	-0.7946			0.8899
	No steam-Hot air drying vs. Steam-Hot air drying	-0.7615			0.9089
	No steam-Hot air drying vs. Steam-Freeze drying	-0.9323			0.7901
	No steam-Hot air drying vs. Steam-Microwave drying	-0.8210			0.8732
	No steam-Freeze drying vs. No steam-Microwave drying	7.284			<0.0001
	No steam-Freeze drying vs. Steam-Sun drying	7.071			<0.0001
	No steam-Freeze drying vs. Steam-Hot air drying	7.104			<0.0001
	No steam-Freeze drying vs. Steam-Freeze drying	6.934			<0.0001
	No steam-Freeze drying vs. Steam-Microwave drying	7.045			<0.0001
	No steam-Microwave drying vs. Steam-Sun drying	-0.2123			>0.9999
	No steam-Microwave drying vs. Steam-Hot air drying	-0.1791			>0.9999
	No steam-Microwave drying vs. Steam-Freeze drying	-0.3500			0.9988
	No steam-Microwave drying vs. Steam-Microwave drying	-0.2387			>0.9999
	Steam-Sun drying vs. Steam-Hot air drying	0.03313			>0.9999
	Steam-Sun drying vs. Steam-Freeze drying	-0.1378			>0.9999
	Steam-Sun drying vs. Steam-Microwave drying	-0.02646			>0.9999
	Steam-Hot air drying vs. Steam-Freeze drying	-0.1709			>0.9999
	Steam-Hot air drying vs. Steam-Microwave drying	-0.05959			>0.9999
	Steam-Freeze drying vs. Steam-Microwave drying	0.1113			>0.9999
Parishin L	No steam-Sun drying vs. No steam-Hot air drying	0.4984	One-Way ANOVA Tukey's post hoc tests	F (7, 16) = 17.71	0.9805
	No steam-Sun drying vs. No steam-Freeze drying	-0.7093			0.8876
	No steam-Sun drying vs. No steam-Microwave drying	-2.004			0.0343
	No steam-Sun drying vs. Steam-Sun drying	-2.216			0.0163
	No steam-Sun drying vs. Steam-Hot air drying	-1.851			0.0581
	No steam-Sun drying vs. Steam-Freeze drying	-4.563			<0.0001
	No steam-Sun drying vs. Steam-Microwave drying	-2.833			0.0018
	No steam-Hot air drying vs. No steam-Freeze drying	-1.208			0.3967
	No steam-Hot air drying vs. No steam-Microwave drying	-2.502			0.0059
	No steam-Hot air drying vs. Steam-Sun drying	-2.714			0.0028
	No steam-Hot air drying vs. Steam-Hot air drying	-2.349			0.0101
	No steam-Hot air drying vs. Steam-Freeze drying	-5.062			<0.0001
	No steam-Hot air drying vs. Steam-Microwave drying	-3.332			0.0003
	No steam-Freeze drying vs. No steam-Microwave drying	-1.294			0.3196
	No steam-Freeze drying vs. Steam-Sun drying	-1.506			0.1761
	No steam-Freeze drying vs. Steam-Hot air drying	-1.141			0.4619
	No steam-Freeze drying vs. Steam-Freeze drying	-3.854			<0.0001
	No steam-Freeze drying vs. Steam-Microwave drying	-2.124			0.0225
	No steam-Microwave drying vs. Steam-Sun drying	-0.2120			>0.9999
	No steam-Microwave drying vs. Steam-Hot air drying	0.1529			>0.9999
	No steam-Microwave drying vs. Steam-Freeze drying	-2.560			0.0048

	No steam-Microwave drying vs. Steam-Microwave drying	-0.8297			0.7888
	Steam-Sun drying vs. Steam-Hot air drying	0.3649			0.9969
	Steam-Sun drying vs. Steam-Freeze drying	-2.348			0.0102
	Steam-Sun drying vs. Steam-Microwave drying	-0.6177			0.9406
	Steam-Hot air drying vs. Steam-Freeze drying	-2.712			0.0028
	Steam-Hot air drying vs. Steam-Microwave drying	-0.9826			0.6315
	Steam-Freeze drying vs. Steam-Microwave drying	1.730			0.0870
Parishin R	No steam-Sun drying vs. No steam-Hot air drying	0.6401	One-Way ANOVA Tukey's post hoc tests	F (7, 16) = 7.304	0.9566
	No steam-Sun drying vs. No steam-Freeze drying	-0.2909			0.9996
	No steam-Sun drying vs. No steam-Microwave drying	0.1418			>0.9999
	No steam-Sun drying vs. Steam-Sun drying	-2.178			0.0382
	No steam-Sun drying vs. Steam-Hot air drying	-1.653			0.1806
	No steam-Sun drying vs. Steam-Freeze drying	-2.108			0.0475
	No steam-Sun drying vs. Steam-Microwave drying	-1.916			0.0852
	No steam-Hot air drying vs. No steam-Freeze drying	-0.9310			0.7756
	No steam-Hot air drying vs. No steam-Microwave drying	-0.4983			0.9888
	No steam-Hot air drying vs. Steam-Sun drying	-2.818			0.0049
	No steam-Hot air drying vs. Steam-Hot air drying	-2.293			0.0265
	No steam-Hot air drying vs. Steam-Freeze drying	-2.748			0.0061
	No steam-Hot air drying vs. Steam-Microwave drying	-2.556			0.0114
	No steam-Freeze drying vs. No steam-Microwave drying	0.4327			0.9951
	No steam-Freeze drying vs. Steam-Sun drying	-1.887			0.0930
	No steam-Freeze drying vs. Steam-Hot air drying	-1.362			0.3721
	No steam-Freeze drying vs. Steam-Freeze drying	-1.817			0.1141
	No steam-Freeze drying vs. Steam-Microwave drying	-1.625			0.1947
	No steam-Microwave drying vs. Steam-Sun drying	-2.319			0.0244
	No steam-Microwave drying vs. Steam-Hot air drying	-1.795			0.1215
	No steam-Microwave drying vs. Steam-Freeze drying	-2.250			0.0304
	No steam-Microwave drying vs. Steam-Microwave drying	-2.058			0.0554
	Steam-Sun drying vs. Steam-Hot air drying	0.5246			0.9850
	Steam-Sun drying vs. Steam-Freeze drying	0.06985			>0.9999
	Steam-Sun drying vs. Steam-Microwave drying	0.2615			0.9998
	Steam-Hot air drying vs. Steam-Freeze drying	-0.4547			0.9935
	Steam-Hot air drying vs. Steam-Microwave drying	-0.2631			0.9998
	Steam-Freeze drying vs. Steam-Microwave drying	0.1917			>0.9999
